# Evaluation of Incisional Wound Healing in Dogs after Closure with Staples or Tissue Glue and Comparison to Intradermal Suture Pattern

**DOI:** 10.3390/ani13030426

**Published:** 2023-01-27

**Authors:** Dimitrios B. Balomenos, Pagona G. Gouletsou, Apostolos D. Galatos

**Affiliations:** 1 Clinic of Surgery, Faculty of Veterinary Science, School of Health Sciences, University of Thessaly, 43100 Karditsa, Greece; 2 Clinic of Obstetrics and Reproduction, Faculty of Veterinary Science, School of Health Sciences, University of Thessaly, Trikalon 224, 43100 Karditsa, Greece

**Keywords:** canine, staples, tissue adhesive, glue, intradermal, poliglecaprone 25, wound healing

## Abstract

**Simple Summary:**

The choice of suture material for skin closure can affect the final cosmetic outcome, the risk of wound infection and other complications in companion animals. We assessed two quickly and easily applicable skin closure methods, staples and tissue glue, and compared them to intradermal suturing in dogs, by using cosmetic, clinical, ultrasonographical and histological evaluation. The results indicate that glue had a less favorable outcome and that intradermal suture was the best, however not significantly better than staples, which are applied easier and in significantly less time.

**Abstract:**

The study aimed to monitor the healing process in the canine skin following surgical incision and closure using staples or tissue glue and to compare them with the intradermal suture pattern. Surgically created skin incisions in 10 dogs were apposed with staples, tissue glue (n-butyl cyanoacrylate) and continuous intradermal pattern. The cosmetic appearance of the wounds was blindly evaluated on days 7, 14 and 28 and once a month until the end of the experiment, i.e., one year after the incision. Ultrasonographic and clinical evaluation was performed on days 0–10, 12, 14, 16, 18, 21, 24 and 28, once a week until the end of the 3rd month and once a month until the end of the experiment. Histopathological evaluation was performed on days 7, 14, 28, 180 and 365. The median time required for the performance of each technique differed significantly between techniques; stapling lasted 21 s, glue 2 min 16 s and intradermal 15 min 37 s. Cosmetic appearance with glue was statistically worse than staples and intradermal. The clinical appearance of intradermal was significantly better than glue and staples. No significant differences were found at histological evaluation; however, glue had the worst score throughout the experiment. The overall evaluation of the techniques showed that glue had the worst score compared to intradermal and staples, with the difference being statistically significant in the first postoperative week. Intradermal suture pattern is much better than glue application for skin closure in dogs, whilst is not significantly better than staples. Staples should be preferred when time is an important factor.

## 1. Introduction

Most surgical wounds are typically treated by first intention healing with the use of sutures. Continuous intradermal skin closure has become increasingly popular in surgical procedures in small animals [1,2,3,4,5,6] since it has many advantages, including decreased scar formation, no need for suture removal, and reduction of tissue inflammation and self-induced trauma [6]. However, even if it is considered to be a better option for skin closure [5], continuous intradermal closure is technically demanding and time-consuming [1,2,3,4,5]. Alternative closure techniques or materials, such as tissue adhesives (glue) and skin staples, have been used mainly in humans and are preferred in many cases, since are credited for faster closure and easy application.

Almost all studies in humans and dogs that determine the effects of various techniques used for the closure of surgical wounds focus on the cosmetic appearance and the patient’s and/or surgeon’s satisfaction [3,7,8,9,10,11,12,13,14]. Surgical wound healing assessment often involves scoring of erythema and exudate or infection. More detailed information on the state of the wound has relied on invasive skin biopsy, providing tissue samples that yield objective quantitative data relevant to the healing process, but this inevitably produces further damage that is not acceptable in human or companion animal patients. Cutaneous ultrasonography is a novel technology of skin wound assessment that enables monitoring changes to organs and tissues over time and permits the evaluation, comparison and additional assistance in the clinical interpretation of findings. However, to date, ultrasonography has not been used as a means of studying the healing process of surgical incisions closed by different methods and materials neither in humans nor in other animal species. Furthermore, even if there are a few studies on the use of tissue adhesives on an animal model such as rodents [15] and pigs [16], there is no study on the use of staples or tissue adhesive in dogs.

The aim of the study was to monitor the healing process in the canine skin following surgical incision and closure using skin staples or tissue glue (n-butyl cyanoacrylate) and to compare them with intradermal (continuous intradermal suture pattern with burying of the knot using 4-0 poliglecaprone 25), by cosmetic, clinical, ultrasonographical and histological evaluation. Furthermore, the difficulty and the time required to close each wound were also estimated and compared.

## 2. Materials and Methods

### 2.1. Animal Ethics

The study was performed in the research facility area of the Clinic of Surgery, Faculty of Veterinary Medicine, University of Thessaly. The research protocol of the study was approved by the Greek National Animal Ethics Committee (license number: 1174/13.4.2009), as being in accordance with the European Union legislation concerning the welfare of laboratory animals. Throughout the study, all legal and ethical requirements with regard to the welfare of experimental animals have been met.

### 2.2. Animals

Ten healthy Beagle-breed dogs (5 females, 5 males), aged 1 to 5 years, were used in the study. During the first month of the study, each animal was housed individually; next, animals were separated into three pens, two pens consisting of 3 members and one containing 4. For the entire procedure, strict measures of pain management and handling were taken, while the experiments were according to the laws of the EU.

### 2.3. Inclusion Criteria

All dogs were clinically healthy; no wounds, scars or active skin lesions were evident in the areas where incisions were to be performed. Before the study, the health of each dog was assessed by clinical and laboratory examinations (full haematological and blood and urine biochemical examination); the examinations were repeated at monthly intervals throughout the study period.

The only pharmaceutical or immunological products administered to the animals for 30 days prior to and throughout the study were the following: (a) scheduled vaccines and routine antiparasitic products and (b) antibiotics and opioids, administered during the peri-operative period, as detailed below.

### 2.4. Experimental Design

Dogs were premedicated by intramuscular injection of dexmedetomidine (300 μg/m^2^; Dexdomitor, Pfizer Hellas, Thessaloniki, Greece) and morphine (0.5 mg/kg, Morphina cloridrato, Molteni Pharmaceutici, Florence, Italy). After 30 min, anaesthesia was induced by intravenous administration of thiopental sodium 2.5% (5–7 mg/kg, Pentothal, Abbott Hellas, Alimos, Greece), and, after intubation of the trachea, maintained with isoflurane (1.5–2%, Aerrane, Baxter, Copton, UK) in oxygen (1 L/min), using a semi-closed anaesthesia circuit. During anaesthesia, Lactated Ringer’s solution (10 mL/kg/hour, Lactated Ringer’s, VIOSER, Pelinnei, Greece) was administered by continuous intravenous infusion.

All incisions were created and closed by the same veterinary surgeon (PGG). Three incisions, 12 cm long, were made through the skin and subcutaneous tissue at the lateral aspect of each thigh, which were parallel to the long axis of the femur, and 7 cm apart from each other when two of them were made at the same thigh. After vascular clamping with haemostatic forceps for controlling bleeding, the subcutaneous tissue was closed primarily with a continuous subdermal suture pattern by using 3–0 polyglactin 910 (Vicryl, Ethicon Inc., Somerville, MA, USA). Subsequently, the selected skin closure technique was performed; the technique to be performed was decided randomly on the spot, after selecting a pre-sealed envelope, in which the combination to be performed was mentioned. The procedure was performed initially at the right thigh and subsequently at the left. Skin staples (Aproximate, Ethicon Inc., Somerville, MA, USA) [staples], n-butyl cyanoacrylate tissue glue (Vetbond, 3M) [glue] and continuous intradermal suture pattern with burying of the knots (4-0 poliglecaprone 25; Monocryl, Ethicon Inc., Somerville, MA, USA) [intradermal], were used. The time required to perform each technique was recorded.

In the skin stapling closure method, the staples were placed along the surgical incision in such a way that the distance between them was 8 mm and the distance from the wound edges was 6–7 mm.

In the tissue adhesive application, the n-butyl glue was applied to the apposed wound edges as a thin, single and continuous layer. Care was taken to avoid adhesive pooling or the thick application of glue.

In the intradermal suture pattern (i.e., continuous horizontal mattress in the dermal layer with burying of the knots), the initial knot of suture material was buried in the subcutaneous tissue at a distance of 4 mm from the commissure of the wound, and then the suture material was directed towards the start of the incision in the middle of the dermis. The needle was positioned horizontally, through the dermis, by taking particular care to confirm that no material of the suture crossed the epidermis. Approximately 2.5 cm before the end of the wound, the suture was anchored with a buried knot, so that a biopsy could be performed in the last 2 cm of the suture line, without collapse of the entire suturing. At the last passage of the suture from the dermis, the needle was directed backwards and the suture was fixed with an Aberdeen knot in the subcutaneous tissue. After knot formation, the tip of the suture material was directed towards the subcutaneous tissue, at the lateral side of the incision and finally outside the skin; that way, the knot was moved away from the skin healing layer. The suture material was cut at the level of the skin surface.

The time required to complete each closure was recorded by a stopwatch.

### 2.5. Postoperative Care

Immediately after the surgery and until the 10th postoperative day (po.d.), Elisabethan collars were placed on the dogs, and bandages were applied on the thighs to protect the wound site. On the 10th po.d., the staples were also removed.

Dogs received morphine at a dose rate as above, im, every four hours for three days postoperatively and every six hours for the next two days. Moreover, an amoxicillin/clavulanic acid combination was administered to the animals (Synulox, Haupt Pharma Italy, Latina S.r.l., Borgo San Michele, Italy) at the dose rate of 12.5 mg/kg, sc, twice daily until the 10th po.d.

### 2.6. Cosmetic Evaluation

The cosmetic appearance of the wounds was blindly evaluated by two experienced surgeons (PGG and ADG) by assessing wound photographs taken on postoperative days 0–10, 12, 14, 16, 18, 21, 24 and 28, once a week until the end of the 3rd postoperative month and once a month until the end of the experiment, i.e., one year after the incision, based on a 1–10 visual analogue scale (1: excellent cosmetic result, 10: bad cosmetic result). The scores from the two assessors were averaged to generate the total cosmetic appearance score for each wound.

The following supporting information is available for this article:

Figure S1. Photographs of the wounds.

### 2.7. Clinical Evaluation

Clinical evaluation was performed immediately after surgical skin closure (Day 0), every day until the 10th po.d., on the 12th, 14th, 16th, 18th, 21st, 24th and 28th po.d., once a week until the end of the 3rd postoperative month and once a month until the end of the experiment. During clinical evaluation, the following parameters were evaluated, always by the same person (author DBΒ): skin thickening, erythema, scar width, abscessation or inflammation, exudate, comedones, hyperpigmentation, suture or staple loss, cross-scaring and wound dehiscence, according to the scoring system proposed by Gouletsou et al. [5] (Supplementary material Table S1). Skin thickening was assessed by using a tuberculin skin testing ruler (skin calipers) on a fold of the skin that contained the incision line and normal skin. The initial thickness (before incision) was recorded and afterwards this value was deducted from each following measurement to produce skin thickening. If skin thickening, erythema or scar width were uneven along the incision, five measurements were taken and the mean value of the five results was used.

### 2.8. Ultrasonographic Evaluation

B-mode real-time ultrasonographic examination of the skin was performed by means of a real-time ultrasound machine. The ultrasound unit (Longport Digital Scanner [LDS1], Longport International Ltd., Silchester, London, United Kingdom) was fitted with a 50.0 MHz polyvinylidene difluoride transducer incorporated into a probe filled with distilled water and scanned using a digital stepping motor. The ultrasound beam was propagated through an aperture covered with a disposable rubber membrane; a new membrane was used for each wound. The transducer was applied to the wound area using light pressure and coupling gel as a transmission medium. Scans perpendicular to the long axis of the incision and the adjacent intact skin were taken. Four transverse images (a, b, c and d) were taken from each wound, in such a way that the distance between the scans was 2 to 3 cm. Wounds were examined daily until the 10th po.d., on the 12th, 14th, 16th, 18th, 21st, 24th and 28th po.d., once a week until the end of the 3rd month postoperatively and once a month thereafter and until the end of the experiment. Furthermore, a scan was performed at the area where the skin punch biopsy would be performed, just a few minutes before the biopsy. The digitized scans were stored on the associated hard drive and were visualized using a color palette (rainbow). Images were compressed laterally to facilitate viewing of the wound area. The wound area calculations were performed using computer software.

The following supporting information is available for this article:

Figure S2. Ultrasound scans of the wound area.

### 2.9. Histological Evaluation of the Healing Process

Histological evaluation of the healing process was performed on postoperative days 7, 14, 28, 180 and 365 [4,5,17,18,19,20]. Eight-millimeter punch biopsy samples [21] were taken from the incisions (the first, 1 cm away from the lower commissure of the incision; the others, 1.5 cm apart between them, proximally) and were immediately bisected, under magnification, perpendicularly to the incision. The tissue samples were fixed in 10% buffered formalin, stained with hematoxylin and eosin and evaluated according to already existing scales [2,5,22,23].

The following supporting information is available for this article:

Figure S3. Photographs of the histological sections of the wound area.

The following parameters were evaluated: necrosis, oedema, inflammation, epithelial gap, presence of suture and tissue reaction around the suture, epithelial thickness, scar width, collagen synthesis, presence of fibroblasts and angiogenesis. The scoring system is presented in Supplementary material Table S2.

### 2.10. Statistical Analysis

All the continuous variables were tested against the normal distribution with the Kolmogorov–Smirnov one-sample test of normality. According to the results of the previews test, the qualitative data are presented as the median (interquartile range) and the quantitative as the frequency and proportion.

For the initial detection of differences between the results of every technique, the Kruskal–Wallis test was used. When the test produced statistically significant results (*p* < 0.05), we performed pairwise comparisons with the Mann–Whitney test, reducing accordingly the significance level with the use of Bonferroni correction.

For intra-group analysis of repeatedly measured variables (separate time intervals), Friedman analysis of variance by ranks was chosen, accompanied by the Wilcoxon for pairwise comparisons, reducing the significance level, following the Bonferroni correction. The level of statistical significance for all comparisons in this study was set at 5%, and all the calculations and tests were performed with IBM SPSS 20 software. (IBM, New York, NY, USA)

An initial exploratory analysis was conducted to examine the fitting of the quantitative data to the normal distribution, with the use of the Shapiro–Wilk test [24]. To compare the differences between the techniques over time, we formulated the Friedman two-way analysis by ranks dividing the experimental period into 5 time periods. The periods were chosen to reflect the different phases of wound healing:

- Time period A (1st–8th po.d.), when inflammation, debridement, and proliferation take place;
- Time period Β (9th–21st po.d.), when proliferation takes place;
- Time period C (22nd–63rd po.d.), when the early stage of maturation takes place;
- Time period D (64th–180th po.d.), when the median stage of maturation takes place;
- Time period E (181st–365th po.d.), when the late stage of maturation takes place.

In order to find a representative (total) score for the cosmetic, clinical, ultrasonographical and histological evaluation, at each time period, separate scores of some of the parameters of each category were added.

For cosmetic examination, intra-observer variability was first examined with the Mann–Whitney U test. Afterwards, the total score was calculated by averaging the separate scores given by the two observers.

For total clinical evaluation, the scores of erythema, skin thickening, scar width, hyperpigmentation, suture or staple loss, cross-scaring and inflammation were added. The values of the erythema, skin thickening and scar width, before being added, were transformed to a four-scale score (0–3), based on the 25th, 50th and 75th percentile of the distribution of the total values.

For the ultrasonographic evaluation, four different tomographic sections (a, b, c and d) were examined at four sites along the scar in each incision, “a” being proximally and “d” being distally to the hip joint. The wound area was calculated for each tomographic plan. The difference in the volumes between the tomographic sections was tested with Wilcoxon Signed Ranks Test, due to salient violation of the normal distribution. Furthermore, the mean u/s wound area of each wound (derived by the four-volume values taken in each wound) was transformed to a four-scale score (0–3), based on the 25th, 50th and 75th percentile of the distribution of the total values.

For total histological evaluation, the scores of oedema and inflammatory reaction were added. The values of the thickness of the epidermis at the area of wound healing, the epithelial gap and the scar width, before summation, were transformed to a four-scale score (0–3), based on the 25th, 50th and 75th percentile of the distribution of the total values. Tissue necrosis was not included, as there was no sign in any of the samples, and no further analysis was conducted. Collagen deposition, fibroblast presence and angeiogenesis were neither included in the summation, as they did not differ between the techniques.

Furthermore, in order to find if there is any correlation between u/s estimated mean wound areas and various histological and clinical parameters, Spearman’s rank order correlation coefficient was used.

Finally, to compare the techniques over time, the total representative score of each technique was evaluated in each time period, by adding each time the cosmetic, clinical, ultrasonographical and histological total scores for the particular period.

## 3. Results

### 3.1. Technique Duration

The time required for each skin closure technique is shown in Table 1; from the comparison, we can conclude that they all differed significantly (*p* < 0.001).

### 3.2. Cosmetic Evaluation

There was no statistically significant difference between the scores awarded by each of the two assessors during the evaluation of the cosmetic appearance of the wounds (*p* = 0.91). Moreover, no statistical difference was observed between the median scores awarded by each of the two assessors in accord with the technique employed. No significant differences were observed between the techniques till period E, whilst in period E statistically significant differences were observed between glue and intradermal (*p* < 0.001).

**Figure 1:**
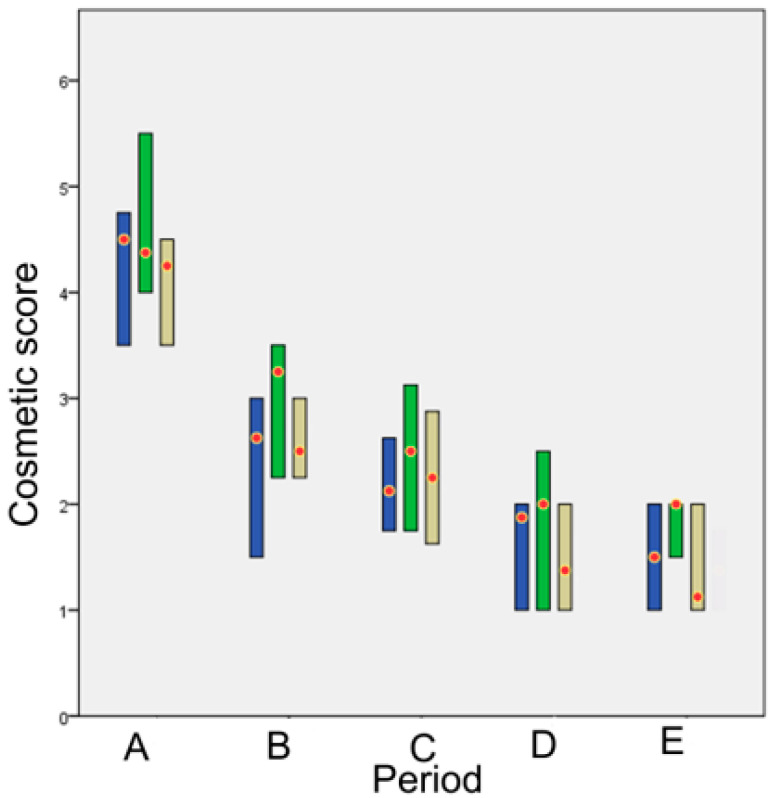
Median score (red dot) and interquartile range (column) of the total cosmetic score for each technique (blue: staples, green: glue, gray: intradermal) in each time period. [period A: 1–8 po.d.; period Β: 9–21 po.d.; period C: 22–63 po.d.; period D: 64–180 po.d.; period E: 181–365 po.d.]. Differences were statistically significant between glue and intradermal in Period E (*p* < 0.001).

The cosmetic score for each technique in each time period is presented in Figure 1.

### 3.3. Clinical Evaluation

#### 3.3.1. Skin Thickening

**Figure 2:**
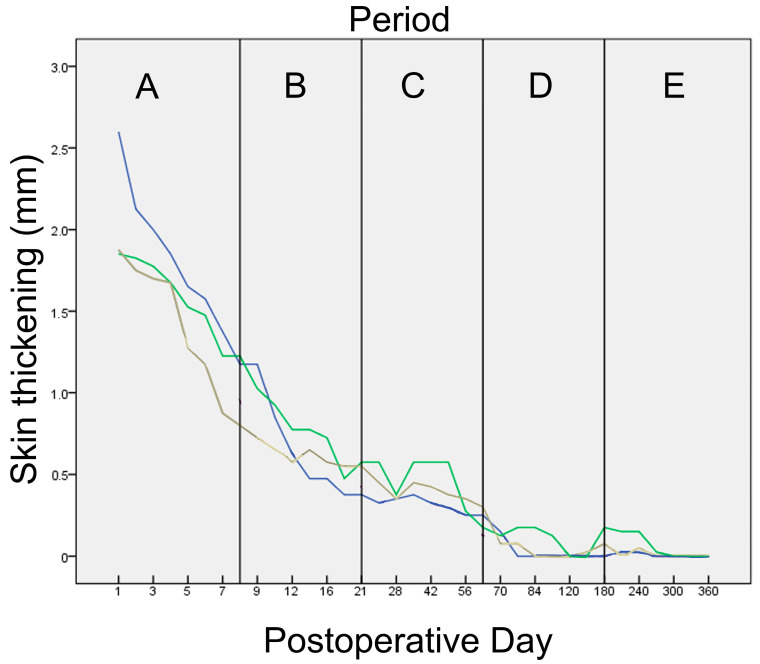
Median skin thickening (in mm) with each technique (blue: staples, green: glue, gray: intradermal) at each time point.

**Figure 3:**
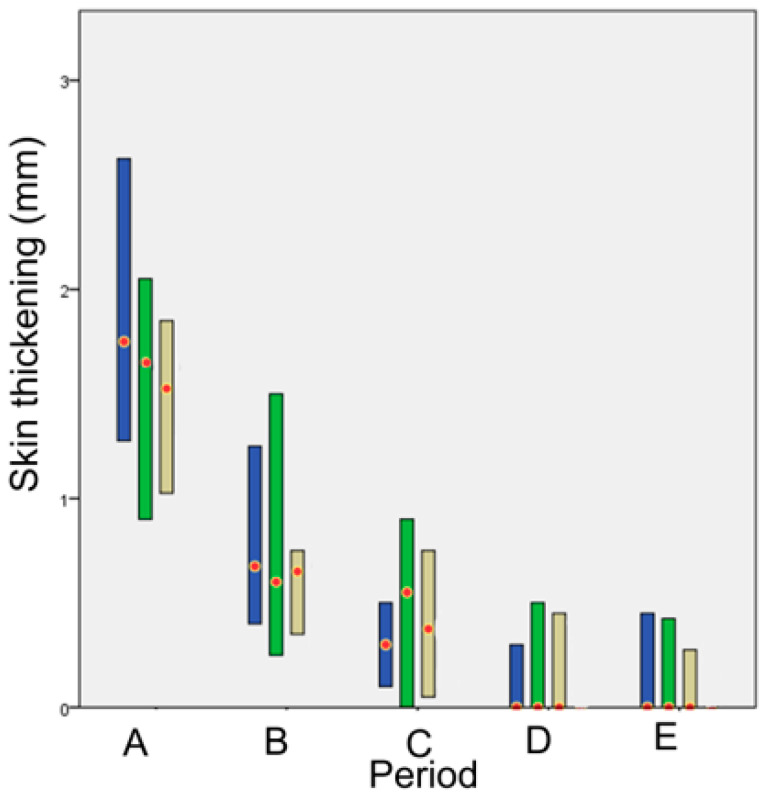
Median (red dot) and interquartile range (column) of skin thickening (in mm) for each technique (blue: staples, green: glue, gray: intradermal) in each time period. [period A: 1–8 po.d.; period Β: 9–21 po.d.; period C: 22–63 po.d.; period D: 64–180 po.d.; period E: 181–365 po.d.]. In period A, the skin thickening as regards staples was significantly different from intradermal (*p* < 0.001).

Skin thickening at the wound area was observed from the 1st po.d., when the largest skin thickening was observed with all techniques and in all animals, due to the swelling of the tissues. The thickening was more intense the first po.d. week with all techniques. Afterwards, it decreased gradually and subsided completely around the 70th po.d. with all techniques. The median skin thickening for each technique at each time point is presented in Figure 2. In period A, the skin thickening regarding staples was significantly bigger than intradermal (*p* < 0.001). No significant differences were observed between the techniques at any other time period (Figure 3).

#### 3.3.2. Erythema

**Figure 4:**
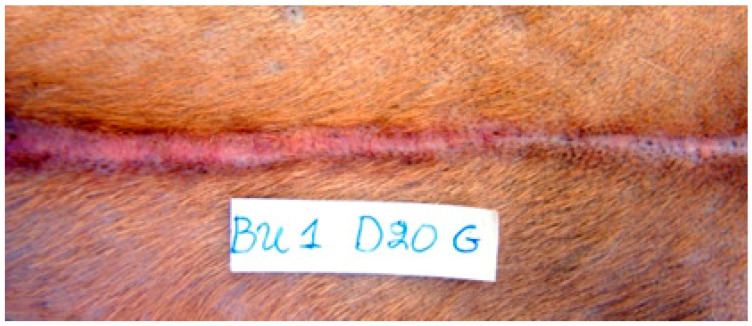
Erythema at the wound area closed with glue 20 days postoperatively.

Erythema was observed in all animals with all techniques during period A. In period B, it remained in some animals where glue (Figure 4) and staples were used. In period C, it was only observed in one incision closed with staples and in one closed with glue. No significant differences were revealed between the techniques in any time period.

#### 3.3.3. Scar Width

**Figure 5:**
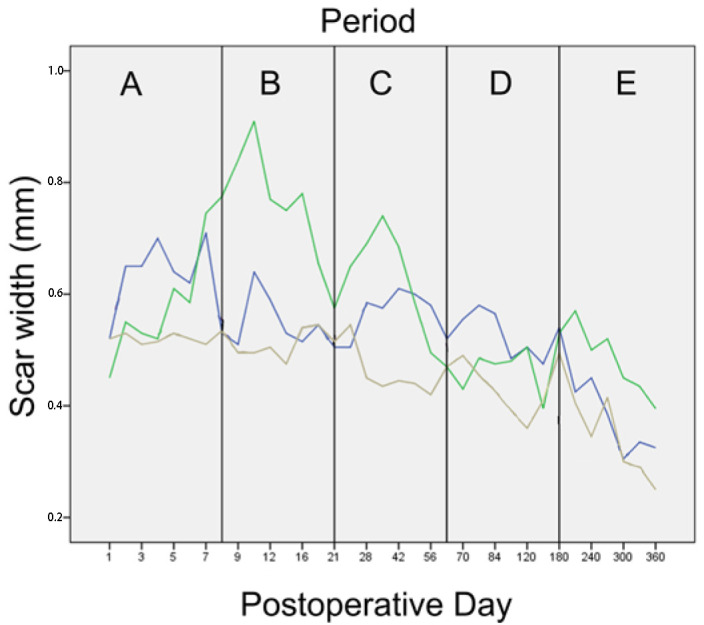
Median scar width (in mm) for each technique (blue: staples, green: glue, gray: intradermal) at each time point. [period A: 1–8 po.d.; period Β: 9–21 po.d.; period C: 22–63 po.d.; period D: 64–180 po.d.; period E: 181–365 po.d.].

**Figure 6:**
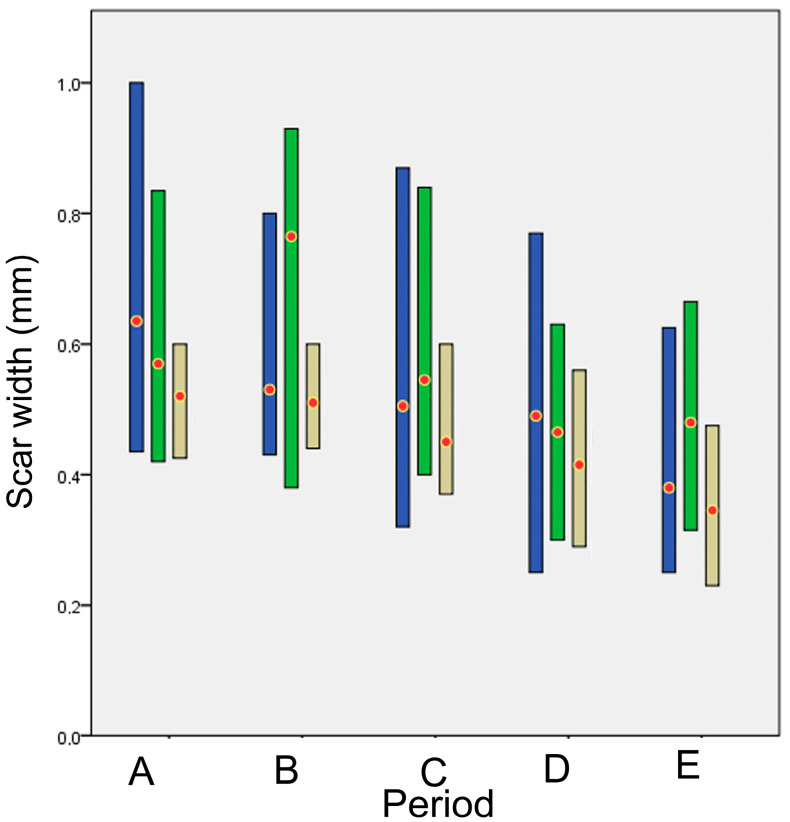
Median (red dot) and interquartile range (column) of scar width (in mm) for each technique (blue: staples, green: glue, gray: intradermal) in each period. [period A: 1–8 po.d.; period Β: 9–21 po.d.; period C: 22–63 po.d.; period D: 64–180 po.d.; period E: 181–365 po.d.]. In periods B, C and E, the median scar width with glue was significantly wider from intradermal (*p* = 0.001, *p* = 0.008 and *p* < 0.001, respectively).

The median scar width with each technique at each time point is presented in Figure 5. The scar width decreased gradually until the 21st po.d. with all techniques but glue, with which the scar width increased. Then, up to the 180th po.d., it remained stable with all techniques but glue, with which it decreased. Until the 365th po.d., the scar width decreased with all techniques. The last measurement of the scar width, taken one year post-operatively, showed a wider scar for glue. The scar width (in mm) in each period is presented in Figure 6. In periods B, C and E, the scar width with glue was significantly wider than with intradermal (*p* = 0.001, *p* = 0.008 and *p* < 0.001, respectively).

#### 3.3.4. Inflammation and Abscessation

Inflammation was observed in six animals (some in more than one incision), and in all but one wound was evaluated with a score of 1, i.e., mild inflammation. In only one case a microabscess was noticed on the 28th po.d. at the wound sutured with intradermal. The number of inflammation cases observed for each technique is shown in Table 2. There was no statistically significant difference in the number of inflammation incidents observed between techniques.

#### 3.3.5. Comedones

Comedones were observed only on three occasions in two animals. One was observed on the 77th po.d. in an incision closed with staples and the other two in an incision closed with intradermal on the 35th and 42nd po.d. No significant difference was found between the techniques.

#### 3.3.6. Hyperpigmentation

**Figure 7:**
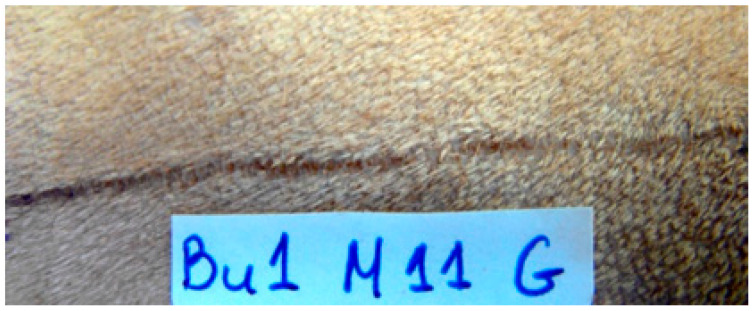
Hyperpigmentation at wound area closed with glue 11 months postoperatively.

Hyperpigmentation was observed in all wounds with all the techniques from the 28th (or 35th) until the 365th po.d. The intensity of the hyperpigmentation differed according to the technique and the period of evaluation. Wounds with intense hyperpigmentation initially and with mild hyperpigmentation afterwards were observed with staples. From the 12th po.d., hyperpigmentation was also observed in the cross-scaring formations. With glue, the wounds had intense hyperpigmentation that was maintained for a longer period (Figure 7) when compared with intradermal closure in which hyperpigmentation was absent or mild and remained for a shorter period. In periods C and D, staples had a significantly greater hyperpigmentation score than intradermal closure (*p* = 0.003 and *p* < 0.001, respectively), and glue had a significantly greater hyperpigmentation score than intradermal closure in periods C, D and E (*p* = 0.003, *p* < 0.001 and *p* < 0.002, respectively).

#### 3.3.7. Suture or Staple Loss and Wound Dehiscence

During the first 10 po. days, only four staples were removed from the skin by the animals. One staple was removed on the 1st po.d. by one animal, two on the 4th po.d. by another and one on the 7th po.d. by a third animal. Only one case of wound dehiscence was observed in an incision closed with staples. This occurred on the 10th po.d., two days after staple removal; its length was 0.8 cm, and its width was 0.5 cm. The defect closed four days afterwards by second intention.

#### 3.3.8. Cross-Scaring Formation

**Figure 8:**
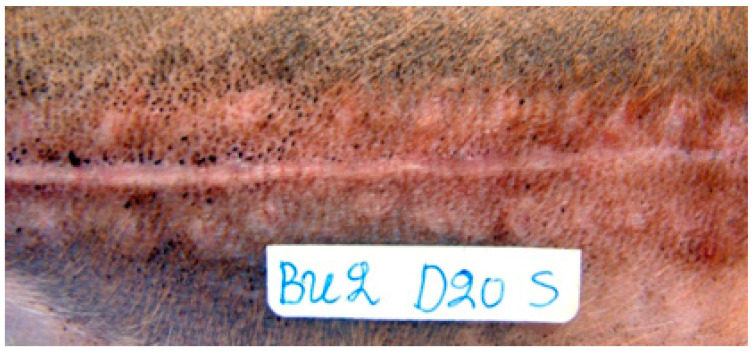
Cross scar formation at wound area closed with staples 20 days postoperatively.

Cross-scaring formations were observed in all incisions closed with staples until the 16th po. day. After this time the incidence of cross-scaring decreased over time. On the 21st po.d., cross-scaring was observed in 9/10 scars, in 7/10 scars on the 28th day, 5/10 on the 35th day, 3/10 on 395 the 42nd day and 1/10 scars on the 70th po.d. (Figure 8).

#### 3.3.9. Total Clinical Evaluation

**Figure 9:**
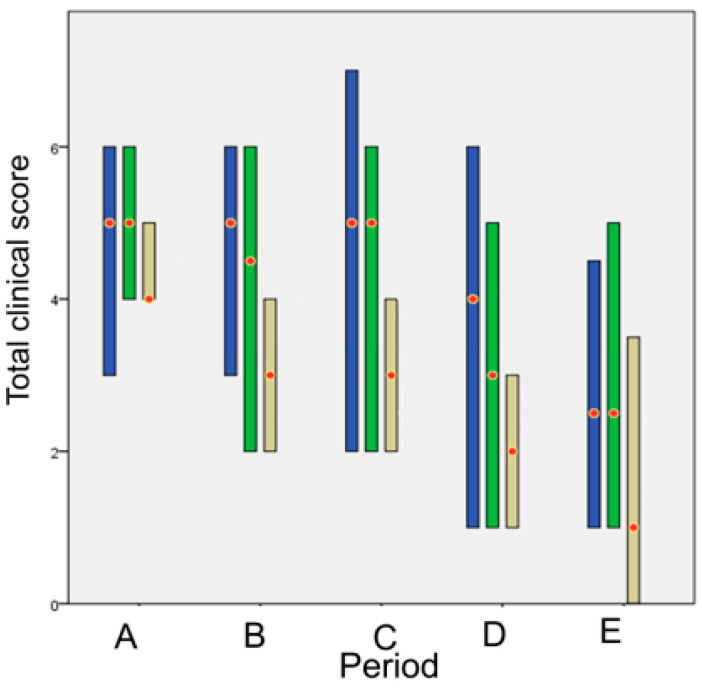
Median (red dot) and interquartile range (column) of total clinical evaluation score for each technique (blue: staples, green: glue, gray: intradermal) in each period. [period A: 1–8 po.d.; period Β: 9–21 po.d.; period C: 22–63 po.d.; period D: 64–180 po.d.; period E: 181–365 po.d.]. In period B, staples have a significantly worse score than glue (*p* = 0.004) and intradermal (*p* < 0.001).

The score of the total clinical evaluation for each technique in each period is shown in Figure 9. No statistically significant difference was found in all periods except period B, where staples were found to have significantly worse scores than glue (*p* = 0.004) and intradermal (*p* < 0.001).

### 3.4. Ultrasonographic Evaluation

**Figure 10:**
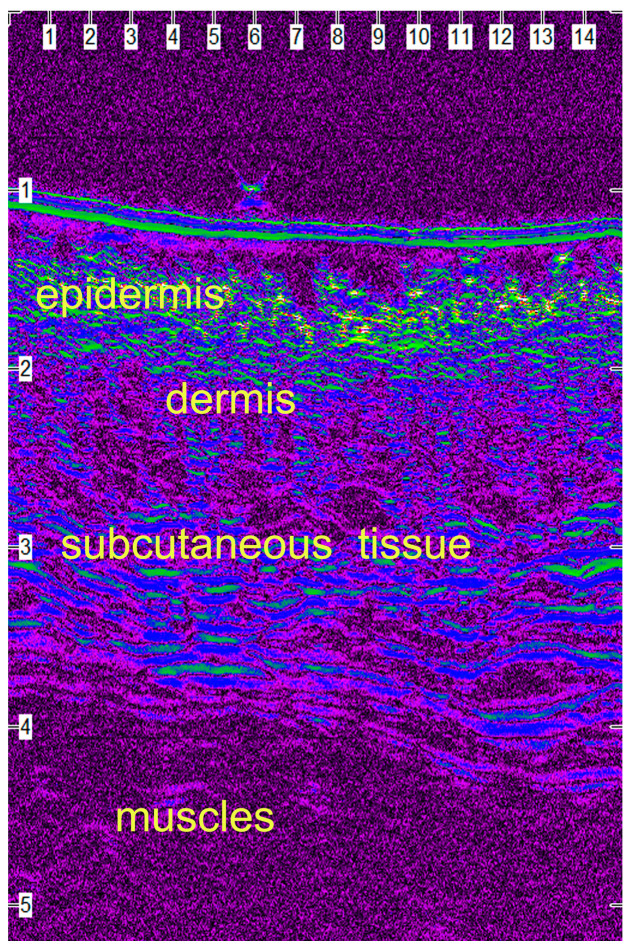
Ultrasonographic images of the normal canine skin of the lateral thigh. The epidermis, dermis and subcutaneous tissue are clearly visible. The digitized scans are visualized using a color palette (rainbow) and are compressed laterally to facilitate viewing of the wound area (scale is in mm).

Before the skin incision, in all ultrasound scans the epidermis was clearly visible as an hyperechoic linear layer. The dermis had a granular echotexture that appeared to become more linear in the deeper parts. Subcutaneous tissue was recognized at a greater depth, as a thicker layer characterized by an inhomogeneous hypoechoic or non-echogenic pattern (Figure 10).

**Figure 11:**
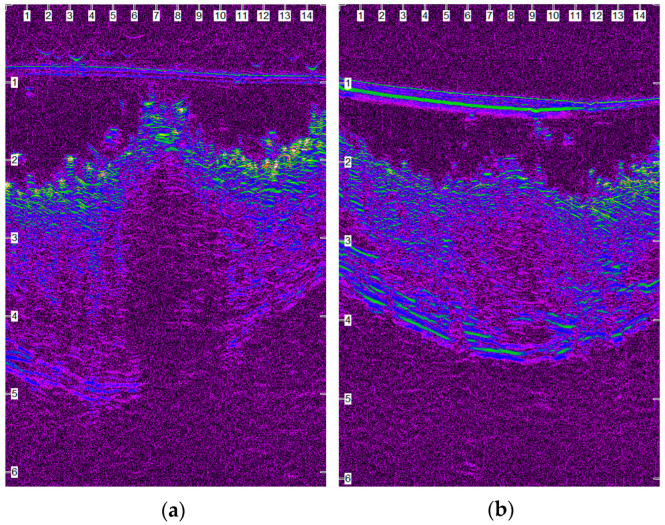
Ultrasonographic images of the wound area closed with glue 4 days (**a**) and intradermal 84 days (**b**) postoperatively. The image is compressed laterally to facilitate viewing (scale is in mm).

After wound closure, the ultrasonographic image of the skin in the wound area differed from the adjacent normal one. The shape of the epidermis had been deformed, creating a cone that protruded 1–2 mm. The area of the dermis at the incision site was enlarged and hypoechogenic compared to the normal adjacent one. Specifically, the wound area was ultrasonographically (u/s) defined dorsally by the two echogenic, uplifted epidermal edges, laterally by the two echogenic and thickened dermal edges and ventrally by the anechoic oedematous subcutaneous tissue (Figure 11a). As wound healing proceeded, the wound area size and the tissue oedema diminished, and the progressive collagen deposition altered echo intensity, making wound boundaries more complex but not indistinguishable (Figure 11b).

**Figure 12:**
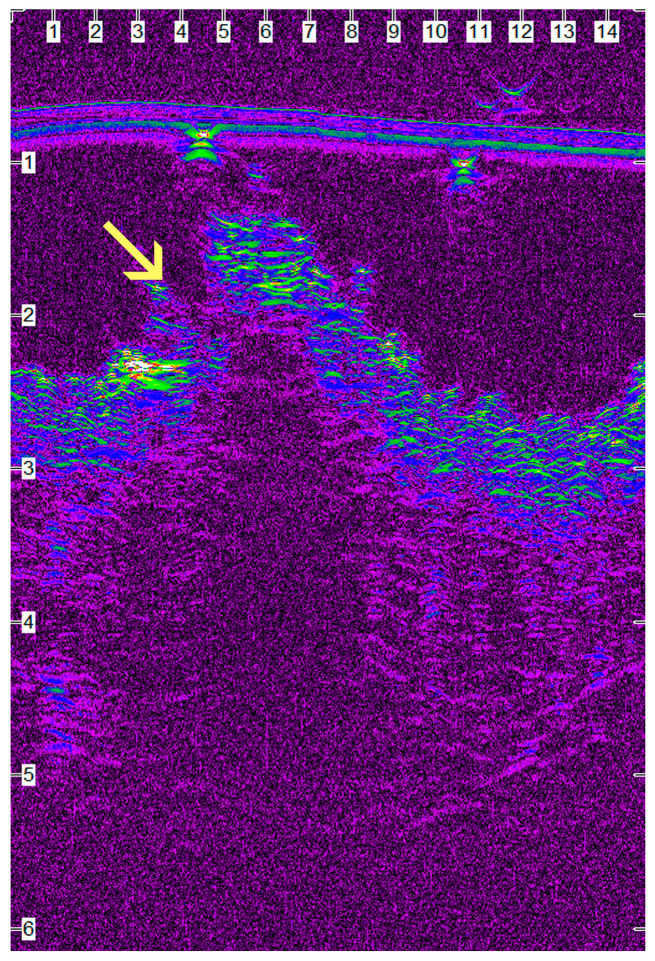
Ultrasonographic image of the wound area closed with glue, on the 9th po.d. The epithelial gap is evident between the epididymal edges at the wound area (arrow). The image is compressed laterally to facilitate viewing (scale is in mm).

Between the 15th po.d. and 42nd po.d., the u/s estimated wound area was reduced to half with all techniques. From the 40th to the 120th po.d., the wound area continued to decrease in size with all techniques. During this period the epidermis at the wound area was losing its conical shape and was becoming flat, and its density approached that of the adjacent normal tissue. The wound area was depicted more clearly with glue and intradermal compared to staples, as with the last technique the wound area was almost isoechoic to the normal adjacent skin. After the 150th po.d., the newly formed mature dermis at the wound area became almost isoechoic to the normal adjacent skin in all incisions with all techniques. In almost all incisions with all techniques, the epidermal edges at the incision site ultrasonographically seemed to be in close contact with each other, from the 1st po.d. until the last day of the experiment. Although it was difficult to monitor the progress of epithelialization with the particular ultrasound scanner, which uses frequencies of 50 MHz, a small epithelial gap was observed during the first postoperative days in some scans of the incisions closed with glue (Figure 12).

**Figure 13:**
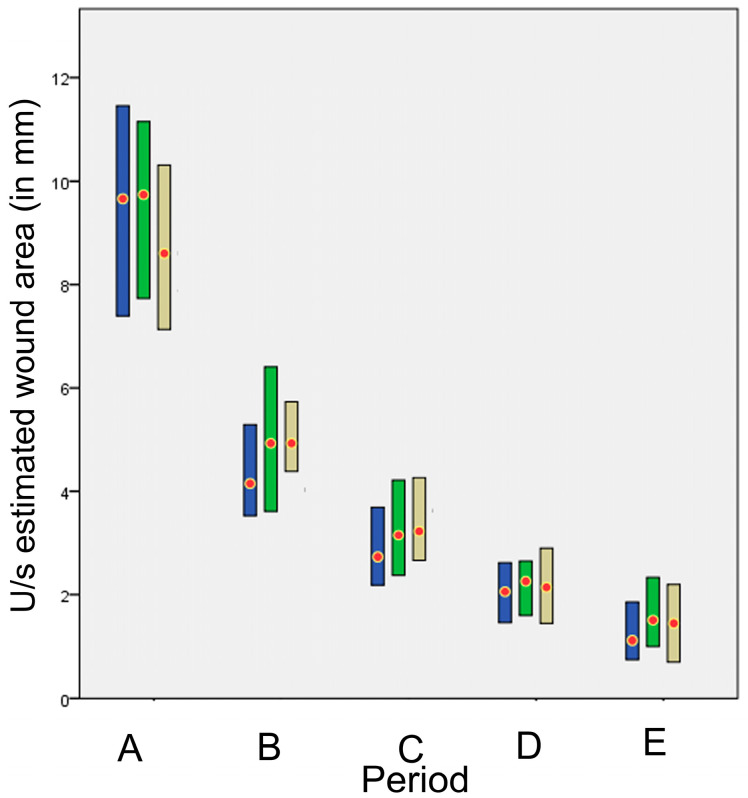
Median (red dot) and interquartile range (column) of u/s estimated wound area (in mm^2^) for each technique (blue: staples, green: glue, gray: intradermal) in each period. [period A: 1–8 po.d.; period Β: 9–21 po.d.; period C: 22–63 po.d.; period D: 64–180 po.d.; period E: 181–365 po.d.]. A statistically significant difference was found in periods B and C between staples and intradermal (*p* = 0.007 and *p* = 0.006, respectively).

The wound area was calculated for each tomographic plan. As the u/s estimated wound area differed significantly between the four different tomography planes of each incision, the mean area was calculated and used for further evaluation. The u/s estimated wound areas in each time period are presented in Figure 13. No statistically significant difference was found between techniques, except for periods B and C, where staples were found to have smaller u/s estimated wound area than intradermal (*p* = 0.007 and *p* = 0.006, respectively).

### 3.5. Histological Evaluation

#### 3.5.1. Necrosis

No necrosis was observed in any tissue examined.

#### 3.5.2. Epithelial Gap

An epithelial gap was observed in three samples of incisions closed with glue on the 7th po.d., with epithelial bridging being completed by the 14th po.d. for all samples. In Beagle No. 1, the width of the gap was 0.4 mm; in Beagle No. 2 it was 0.45 mm; in Beagle No. 3 it was 1.26 mm.

#### 3.5.3. Oedema

In period A, oedema was observed in 4/10 of the samples closed with staples, and it was evaluated with a score of 1; with glue it was observed in 1/10 samples both with a score of 2; with intradermal closure it was observed in 5/10 samples with a score of 1. In period B, oedema was presented only in 1/10 samples with staples and was evaluated with a score of 1. In periods C, D and E, no oedema was observed with any technique.

#### 3.5.4. Inflammation

Skin stapling caused minimal or mild inflammation at the incision area. On the 7th and 14th po.d., infiltration by a small number of neutrophils, macrophages and fibroblasts was observed, which is consistent with the healing process of the wound. Nodular accumulation of macrophages and lymphocytes at the subcutaneous tissue in a few samples was probably caused by the presence of closure material nearby or by traumatic furunculosis. From the 28th po.d. onwards, no inflammation was noticed. It seems that the use of inert metal staples that are removed 10 days po. minimizes foreign body inflammatory reaction in the wound area.

**Figure 14:**
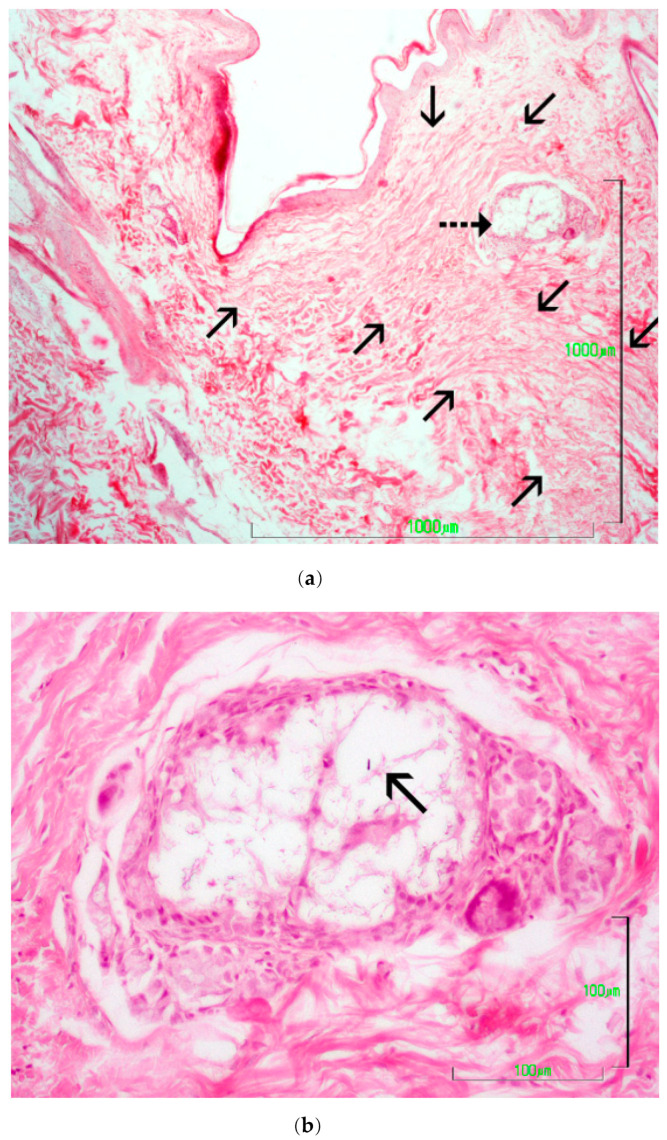
(**a**) Histological section of the wound area (between solid arrows) closed with glue on the 365th po.d. Small quantities of glue deposited below the epidermis (dotted arrow). (**b**) Close-up of the inclusion of glue (arrow) initiating inflammation.

Skin glue application caused mild to medium inflammatory reactions in the wound area. From the 7th to the 28th po.d., the main finding was infiltration by neutrophils, macrophages, lymphocytes and fibroblasts. Some foci with traumatic furunculosis and some comedones were also observed. Afterwards, no inflammation was noticed. The most important finding was that, despite care during the application, small quantities of glue were deposited below the epidermis, initiating inflammation in the dermis, a finding that persisted until the 365th po.d. (Figure 14a,b). The inflammatory reaction around inclusions was of 3–6 layers of cells; however, no necrosis, intense inflammation or purulent drainage was noticed.

With intradermal closure, on the 7th and 14th po.d., the main finding was an infiltration of the wound area by a moderate or large number of neutrophils, macrophages and fibroblasts. From the 28th po.d. onwards, no inflammation was noticed. The inflammatory reaction score is presented in Table 3. No statistically significant difference was observed between the techniques in any period.

#### 3.5.5. Presence of Suture Material and Tissue Reaction

The presence of suture material or its initial position was identified in 24 samples with poliglecaprone 25. Poliglecaprone was not found on the 180th po.d., due to its absorption within 119 days post-implantation. On the 7th, 14th and 28th po.d. the tissue reaction around the suture material was minimal to moderate, and it consisted of macrophages and fibroblasts. In some cases, the suture material was only surrounded by normal skin collagen fibers and not by cells; in other cases, it was surrounded by a small number of macrophages which were arranged in one or two layers, or it was surrounded by more than one layer of inflammatory cells, and these layers were surrounded by fibroblasts and by circular collagen fibers. From 180th po.d. onwards, the area, which the suture material had passed through, was not detected in any tissue sample.

#### 3.5.6. Epithelial Thickness

**Figure 15:**
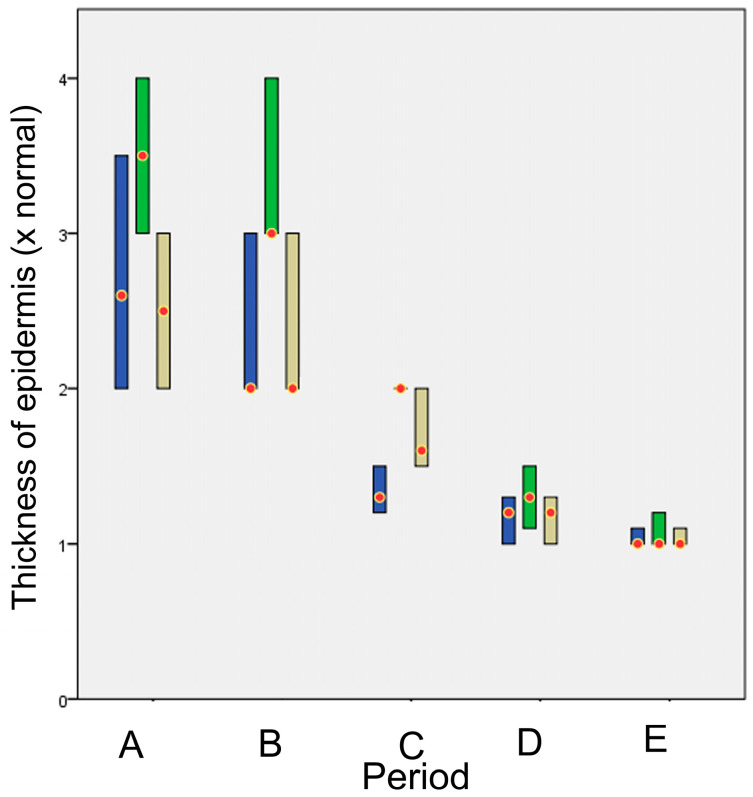
Median (red dot) and interquartile range (column) of the thickness of the epidermis at the wound area (number of times the epidermis thickness is greater than the adjacent healthy epidermis) for each technique (blue: staples, green: glue, gray: intradermal) in each period. [period A: 1–8 po.d.; period Β: 9–21 po.d.; period C: 22–63 po.d.; period D: 64–180 po.d.; period E: 181–365 po.d.]. In period B glue was significantly different from intradermal (*p* = 0.009), and in period C glue differed significantly from staples (*p* < 0.001).

The thickness of the epidermis at the area of wound healing (the number of times the epidermis thickness is greater than the adjacent healthy epidermis) is shown in Figure 15. No significant differences were observed between the techniques, except in period B, where glue was significantly different from intradermal (*p* = 0.009) and period C, where glue differed significantly from staples (*p* < 0.001).

#### 3.5.7. Scar Width

**Figure 16:**
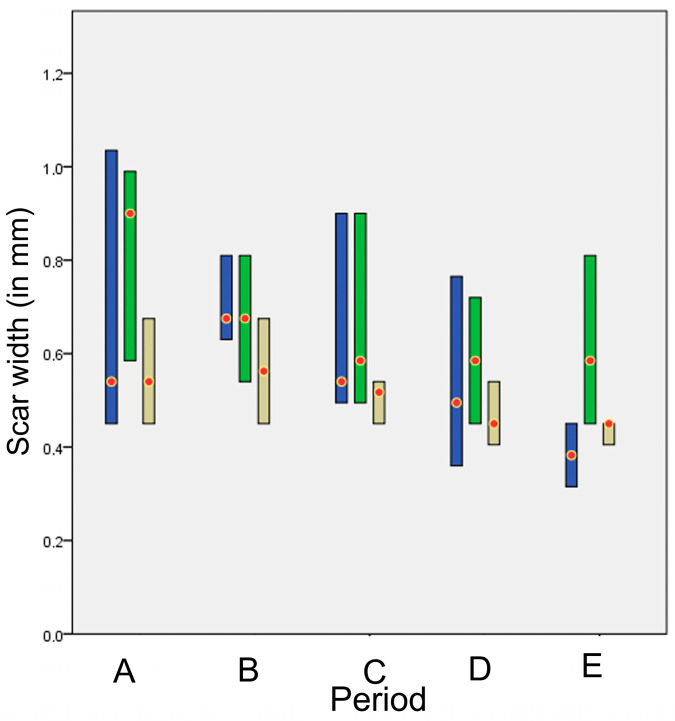
Histologically estimated median (red dot) and interquartile range (column) of the scar width (in mm) for each technique (blue: staples, green: glue, gray: intradermal) in each period [period A: 1–8 po.d.; period Β: 9–21 po.d.; period C: 22–63 po.d.; period D: 64–180 po.d.; period E: 181–365 po.d.]. No statistically significant difference was observed between the techniques (all *p* > 0.043).

The histologically estimated scar width (in mm) for each technique in each period is presented in Figure 16. No statistically significant difference was observed between the techniques (all *p* > 0.043).

#### 3.5.8. Collagen Deposition, Fibroblast Presence, Angiogenesis

The collagen deposition score, fibroblast presence score and angiogenesis score showed no statistically significant differences between the techniques in any period.

#### 3.5.9. Total Histological Evaluation

**Figure 17:**
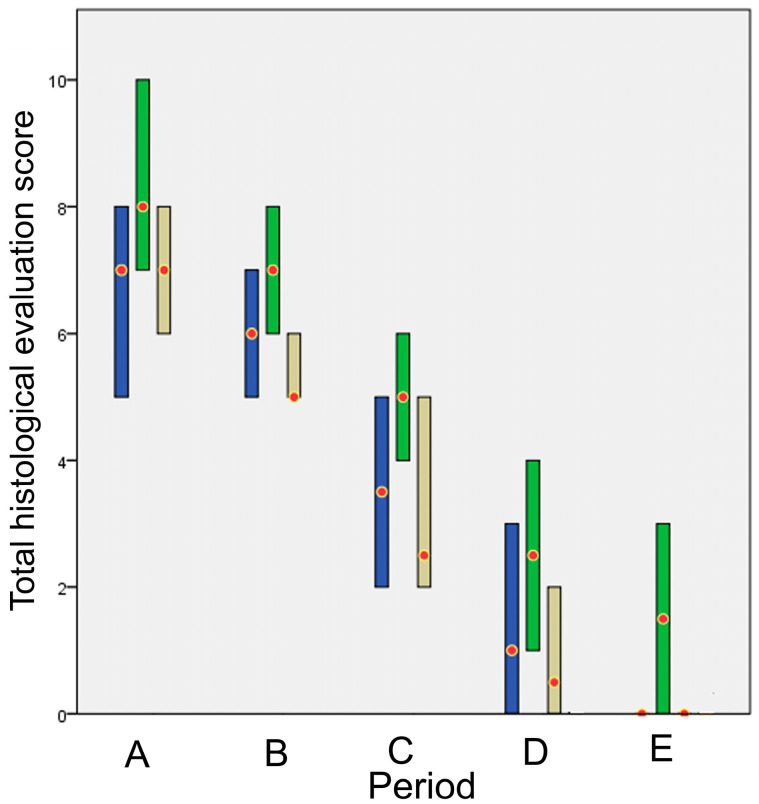
Median (red dot) and interquartile range (column) of the score of total histological evaluation for each technique (blue: staples, green: glue, gray: intradermal) in each period. [period A: 1–8 po.d.; period Β: 9–21 po.d.; period C: 22–63 po.d.; period D: 64–180 po.d.; period E: 181–365 po.d.]. In all periods no significant differences were observed between the techniques (all *p* > 0.043).

The total scores of the histological evaluation in each time period are presented in Figure 17. In all periods no significant differences were observed between the techniques (all *p* > 0.043).

#### 3.5.10. Total Evaluation

**Figure 18:**
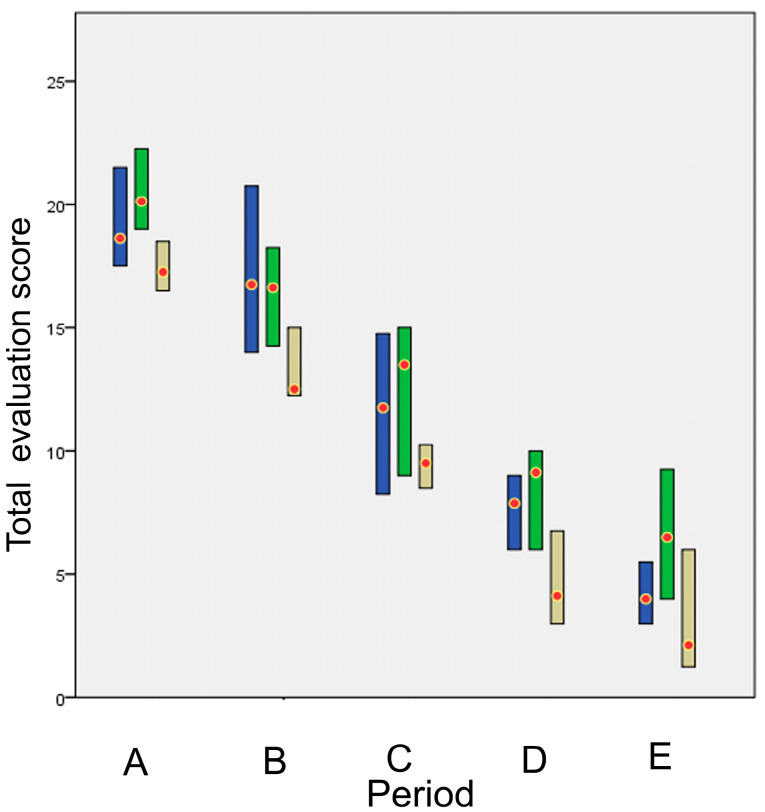
Median (red dot) and interquartile range (column) of total evaluation score for each technique (blue: staples, green: glue, gray: intradermal) in each time period. [period A: 1–8 po.d.; period Β: 9–21 po.d.; period C: 22–63 po.d.; period D: 64–180 po.d.; period E: 181–365 po.d.]. Glue showed a statistically significant difference compared to intradermal in period A, whilst no other statistically significant differences were found between techniques in any other periods.

The total evaluation score is presented in Figure 18. The *p* values of the comparisons between techniques are presented in Table 4. Glue showed a statistically significant difference compared to intradermal in period A, whilst no other statistically significant differences were found between techniques in any other periods.

### 3.6. Correlations

#### 3.6.1. Correlation between u/s Estimated Wound Area and Clinically Evaluated Scar Width

In order to find out if there is any correlation between the u/s estimated wound area and the clinically evaluated scar width, Spearman’s correlation test was used. The test showed that there is a statistically significant positive correlation between the two parameters (r= 0.298, *p* < 0.001); i.e., the larger the u/s estimated wound area, the larger the scar width.

#### 3.6.2. Correlation between u/s Estimated Wound Area and Histologically Estimated Inflammatory Reaction

To examine the association between the u/s estimated wound area at the biopsy site and the histologically estimated inflammatory reaction in the same area, Spearman’s correlation test was used. The test showed that there is a statistically significant positive correlation between the two parameters (r= 0.699, *p* = 0.001); i.e., the larger the u/s estimated wound area, the more intense the inflammatory reaction in the same area.

## 4. Discussion

Various suturing techniques have been described in the literature for their specific benefit in wound closure. It is generally assumed that the intradermal suture pattern has superior cosmetic results, mainly because the epidermis is not penetrated [25], and, therefore, inflammation remains minimal, whilst a fine approximation of wound edges can also be achieved, resulting in minimal scarring [26]. However, although it is considered a better option for skin closure [5], continuous intradermal suture pattern is both technically demanding and time-consuming [1,2,3,4,5]. Alternative closure techniques or materials, such as tissue adhesives and skin staples are credited for faster closure and easier application; however, they have not been tested thoroughly in dogs yet. In our study, the intradermal suture pattern had the best outcome, followed by staples, whilst glue had a significantly worse outcome compared to the first two techniques.

### 4.1. Surgical Operation

As was expected, the application of staples and tissue adhesive was easier in comparison to the intradermal suture pattern. Staples application required significantly less time than tissue glue application and intradermal suture implementation. Tissue glue application required more time compared to staples application and less compared to intradermal suture material implementation, both differences being statistically significant. The successful tissue glue application as a thin and single layer over the apposed wound edges only required the proper eversion of wound edges and the flow control of the adhesive for avoiding deposition into the wound area. However, in two animals, small quantities of glue substance were found inside the wound area, indicating that proper care should be taken during the application, even if this increases the time required. Although the time required for the intradermal suture material implementation is significantly longer in comparison to the other two techniques, this time is not substantial in relation to the overall time of a surgical operation. However, when the time of skin wound closure is an important factor in the emergency setting, in non-stabilized patients, or when the length of the skin to be sutured is considerable, stapling should be the first choice. Chow et al. [27], who performed a systematic review and meta-analysis to compare the performance of tissue glue with standard wound-closure methods used on skin incisions, found that the time for skin wound closure by the majority of studies was considerably shorterwith glue compared to sutures.

### 4.2. Cosmetic Evaluation

In general, the scar appearance for all techniques used was cosmetically evaluated by the assessors with a good rating.

In periods A and B (1st–21st po.d.), the appearance of the incisions as regards intradermal was cosmetically evaluated as better than that of glue and staple, although without a statistically significant difference. The intense erythema and skin thickening observed in the incisions closed with tissue adhesive may have contributed to that rating. In period C (22nd–63rd po.d.), wounds closed with tissue glue still showed erythema, skin thickening and a wide scar, whilst in intradermal suture pattern, mechanical irritation of the wound area by the suture material promoted inflammation, so staples showed a better score. In period D (64th–180th po.d.), the appearance of the incisions with intradermal was cosmetically evaluated as better than for staples and glue, since suture material in intradermal was absorbed around po.d. 110. In period E (181st–365th po.d.), the appearance of the incisions was better with intradermal, followed by staples and then glue. Statistically significant differences were revealed between intradermal and staples in comparison to glue. Consequently, although the cosmetic evaluation of wound area was gradually improving for all techniques, glue showed less favorite cosmetic score than the other techniques from the 9th until the 365th po.d.

In humans, Obermair et al. [28] compared the cosmetic result of the wounds (based on surgeons’ assessment) between the staples application and the intradermal techniques using poliglecaprone 25 and polyglytone 6211 suture materials in gynecological abdominal surgery. The outcome by using staples application was evaluated worst at 1 week and 3 months after surgery compared to the other two groups, whereas there was no difference in surgeons’ assessment at 6 weeks after surgery. Switzer et al. [29] compared the cosmetic outcome of wounds after four weeks in inguinal herniorrhaphy incisions using 2-octylcyanoacrylate tissue adhesive and 4-0 poliglecaprone 25 in a running subcuticular closure and found no significant difference between the techniques. De Graaf et al. [30] compared staples with intradermal suture pattern using 3-0 poliglecaprone 25 for wound closure after caesarean sections. They deduced that the cosmetic evaluation of the incisions in the 6th month was similar between the two techniques. Livesey et al. [31] compared the cosmetic appearance of the scar in surgically closed incisions, performed during total hip replacement, after using tissue adhesive (n-butyl and octyl-blend 70) and staples. No significant difference was observed between the techniques on the 3-month evaluation. Additionally, Chibbaro and Tacconi [32] compared the cosmetic appearance of the scar in long incisions, performed during brain surgery, between tissue adhesive (n-butyl and 2-octyl cyanoacrylates), staples and traditional skin sutures (nylon) and observed no significant difference between the techniques at the 3-month, 6-month and 12-month follow up. Cromi et al. [33] observed a similar cosmetic appearance of the scar 2 and 6 months postpartum, while closing incisions following cesarean section, when compared staples with subcuticular running suture pattern with various suture materials.

### 4.3. Clinical Evaluation

Wound repair by first intention was achieved normally with all techniques, with no severe complications.

#### 4.3.1. Skin Thickening

Skin thickening was observed from the first po.d. in all techniques. The larger skin thickening was observed on the 3rd or 4th po.d. as regards staples and on the 1st po.d. as regards glue and intradermal. Afterwards, skin thickening was decreased at a different degree for each technique until the 63rd po.d.

In period A, skin thickening was larger as regards staples in comparison to glue and intradermal; however, a statistically significant difference was observed only between staples and intradermal. In period B, as the wound healing progressed without complications, skin thickening was decreased for all techniques. Almost from the beginning of this period (10th day), only poliglecaprone 25 suture (intradermal) was still inside the wound area, since the staples had been removed, and the tissue adhesive had been degraded. Concerning the intradermal suture pattern, the skin thickening was observed due to the presence of suture material in the wound at that period. In period C, skin thickness at wound areas in all techniques was even more decreased. In periods D and E, wound skin thickness decreased to pre-wounding skin thickness.

In conclusion, until the 7th pο.d., skin thickening was present with all techniques, but it was more pronounced when staples were placed. Afterwards, until the 21st po.d., skin thickening declined with all techniques, with the incisions closed with staples having the larger decline, especially after staple removal on the 10th po.d. Wound thickness declined even more after the 63rd po.d., returning to almost normal values.

Gouletsou et al. [4,5] observed that skin thickening at the wound area with the intradermal technique, performed with a 4-0 suture, was large on the 1st po.d. and then it decreased gradually until the 20th po.d. On the contrary, when an intradermal suture pattern was performed by using a suture material of a larger diameter (3-0), the skin thickening was increasing from the 1st until the 12th po.d., and then it gradually decreased.

#### 4.3.2. Erythema

Erythema was also observed at the wound area. From the 1st until the 8th po.d., it was more pronounced with staples in comparison to glue and intradermal. From the 9th until the 21st po.d., the extent of erythema decreased in all cases except for one wound closed with staples, where the erythema persisted until the 42nd po.d.

Although erythema is expected during the inflammation stage of the healing process, in this study erythema was mild with all techniques. Gouletsou et al. [4,5] also observed erythema at wound areas in all incisions sutured with intradermal patterns; however, it was more intensive end lasted more, i.e., until the 49th po.d. Gouletsou et al. [4,5] administered no antibiotics post-surgery, in contrast to the present study, and this may have caused a rather prolonged inflammation phase that aggravated erythema. It is also possible that in the present study, the removal of the closure materials on the 10th po.d. might have contributed to the elimination of the erythema. However, the same outcome was also observed with intradermal, although the suture remained in situ until absorption.

#### 4.3.3. Scar Width

An important clinical parameter of the skin healing process is the scar that forms on the skin. On the first po. days, the scar was thin and covered with an eschar (scab). After the eschar had fallen off, the region of the scar was distinguished due to the different texture and because it was colorless and hairless.

In period A, the scar width was smaller with intradermal, probably due to the stronger restraint of wound edges by the suture material present at the wound area. In period B, the scar width decreased with staples, remained small with intradermal and increased considerably with glue. A statistically significant difference was observed between glue and intradermal. The wider scar with glue could not be attributed to wound oedema, since this was moderate with that technique and did not differ from that observed with the other techniques. It is more possible that with tissue adhesives, the glue that attaches only to the epidermal edges cannot probably restrain the dermis, so the scar is widening as healing proceeds. In period C, the scar width was slightly decreased with the staples and intradermal, whilst it was considerably decreased with glue, probably due to wound contraction. A statistically significant difference was observed between glue and intradermal. In period D, no statistically significant differences were observed between techniques. Finally, in period E, the scar width was slightly decreased with all techniques but glue. Statistically significant differences were observed only between glue and intradermal.

Judging from the width of the scar, the intradermal technique was the best during the entire study period due to the narrower scar formed. The staples application had a moderate width scar from the 24th until the 365th po.d., while the application of n-butyl tissue adhesive had the worse outcome because it created a wide scar from the 9th until the 365th po.d. So, if the formation of a narrow scar is desirable, the intradermal suture pattern seems the best solution, whilst staples combine speed and a quite narrow scar.

#### 4.3.4. Abscessation or Inflammation and Exudate

In the present study, few cases of mild inflammation were observed with all techniques, whilst exudate discharge was not observed with any technique. Most of the inflammation cases occurred with intradermal. Inflammation was usually observed in the first postoperative days and lasted 1–4 days, except for one wound sutured with intradermal, where the mild signs of inflammation lasted two months.

In humans, Gatt et al. [34] compared wound infection after suturing skin incisions with staples or with 2-0 polypropylene interrupted vertical mattress sutures or with 3-0 coated polyglactin interrupted vertical mattress sutures, whilst administering antibiotics preoperatively, and observed that wound infection was more intense in the staple and polypropylene groups. Johnson et al. [35] evaluated 242 patients with sternal and saphenous vein harvest wounds, which were closed half way with staples and half with intradermal sutures without burying the knot (polypropylene monofilament suture), and observed that infection was lower with the intradermal suture technique in both locations; however, the difference was not statistically significant. Furthermore, no statistically significant differences in infection were observed by Nagpal et al. [36] on 7th po.d., when comparing cyanoacrylate (n-butyl-) with suture material (3-0 silk percutaneous interrupted sutures) used in the closure of surgical skin wounds. Khan et al. [37] compared skin closure using staples, subcuticular 3-0 absorbable poliglecaprone suture and 2-octyl tissue adhesives in patients undergoing total hip and total knee replacement and observed no statistically significant differences in infection and abscessation (early and late wound complications). According to Iavazzo et al. [38], the use of staples for surgical wound closure was associated with significantly fewer wound infections, compared to the use of sutures in patients undergoing obstetrics or gynecological, general, head/neck and vascular operations, as well as emergency surgical procedures. No statistically significant differences in infection were observed by Eggers et al. [39], who compared four wound closure techniques (staples, 2-octyl- and n-butyl- tissue adhesives and 4-0 poliglecaprone 25) following total knee arthroplasty. Finally, in dogs, Gouletsou et al. [5] observed more severe cases of inflammation and microabscess formation in intradermal suture patterns until the 35th po.d, which, in four cases, were obvious for 3–5 days, whilst in three cases for 15–30 days. This can probably be explained by the fact that no antibacterial drugs were administered postoperatively to these animals. On the contrary, the antibacterial drugs that were given to the animals of the present study, during the first 10 po. days, might have contributed to the low incidence of inflammation. It might be concluded that, even in non-contaminated clear surgical wounds, postoperative administration of antibacterial agents may reduce wound inflammation and prevent microabscess formation, that otherwise might occur.

#### 4.3.5. Comedones

In the present study, comedones were observed on only three occasions in two animals. The first one was with staples, whilst the other two were with intradermal. Comedones have been connected with post-suturing scars in only three studies, i.e., Webster et al. [40], who reported comedones after suturing eyelid skin with catgut, and Gouletsou et al. [4,5], who, 20 to 90 days postoperatively, observed comedones in almost all wounds sutured with intradermal patterns. Smeak [1] suggested that epithelial cysts may form after traumatizing the *stratum basale* of the epidermis or hair follicles during the passage of needle and suture, and Gouletsou et al. [4,5] assumed that this could be an explanation for the presence of comedones at the scars observed in their studies. However, since in the present study only minimal comedones were observed, it should be suggested that probably antibiotics’ administration and elimination of inflammation might also play a role in their absence.

#### 4.3.6. Hyperpigmentation

The alteration of scar color was another characteristic of skin healing in the present study. In the majority of animals with all techniques, from the 35th until the 180th po.d., and in some animals until the 365th po.d., the scar acquired a darker color than that of the adjacent skin. Afterwards, the scar’s color became lighter and did not differ from the color of the adjacent normal skin.

According to Swaim and Henderson [41] and Hosgood [42], the first signs of pigment deposition in the skin become visible 1–2 weeks postoperatively, although the maximum concentration of melanocytes is observed several months later. Melanogenesis depends on both exogenous and endogenous agents, such as alpha melanocyte-stimulating hormone and adrenocorticotrophic hormone, vitamin D3, interleukins or other cytokines, which influence the process of melanin production from melanocytes [43,44]. The change in the scar’s color in our study was observed approximately on the 4th postoperative week, probably due to the action of produced cytokines, which stimulated the production of melanin [45,46]. Afterwards, the color of most scars became normal, except for a few in which it became darker. In this study, the pigmentation intensity of the scars was significantly higher with both staples and glue, while the duration of hyperpigmentation was longer with glue. On the contrary, with intradermal suture pattern, pigmentation was milder and lasted less time. Gouletsou et al. [4,5] also reported hyperpigmentation at the scars after skin suturing. They observed that the scars were becoming darker than the adjacent normal skin from the 28th po.d. until the 180th po.d., and in some cases until the 365th po.d., in most of the wounds sutured with intradermal suture pattern or simple interrupted pattern; however, no statistically significant differences were observed between techniques. In wounds sutured with a simple interrupted pattern, hyperpigmentation was also found at the cross-scaring formations [5], as was the case in the present study with staples application.

#### 4.3.7. Wound Dehiscence

Only one case of wound dehiscence was observed in a wound closed with staples two days after staple removal, and it was 0.8 cm long. Perhaps staples should have been removed some days later so that the tensile strength of the healing wound would have been better. However, no dehiscence occurred to the incisions closed with tissue glue. The use of subcutaneous sutures in the present study might have contributed to this result because some studies report an increased incidence of wound dehiscence when tissue glue alone is used for skin closure [27,47]. In a population control program for cats, Faria et al. [48], when comparing two closure techniques (3-0 nylon simple interrupted sutures and n-butyl-cyanoacrylate tissue adhesive) for ovariohysterectomy, observed no statistically significant difference in wound dehiscence in cats held in a semi-free environment as compared to the confined animals. In humans, Obermair et al. [28], when comparing the staples application and the intradermal technique, by using various suture materials in gynecological abdominal surgery, observed wound dehiscence in six (6/90) patients, two assigned to staples and four assigned to intradermal. Eggers et al. [39], when comparing four wound closure techniques (staples, 2-octyl- and n-butyl- tissue adhesives and intradermal) during total knee arthroplasty, observed dehiscence with slight wound separation (3 mm or less), that was approximately 6% for the adhesive and suture cohorts and 11% for the staples, but without any statistically significant differences.

#### 4.3.8. Cross-Scaring Formation

As expected, cross-scaring formation was observed only when staples were used. The cross-scaring formations were a consistent finding in all animals and were observed immeadiately after staples removal. The cross-scaring formations were intense until the 21st po.d., less pronounced afterwards and almost absent after the 70th po.d. Similar were the findings of Gouletsou et al. [5], who observed cross-scaring formation at the wound area after using a simple interrupted pattern. In their study, cross-scaring formations were present from the 20th to the 180th po.d.; however, in some wounds, they persisted for three years after suturing. Crickeler [49] reported that cross-scaring is probably the result of the stricture of skin inside the stitches’ loop, which induces ischaemia and scar formation. Making the stitches loose helps to eliminate the problem; however, subcutaneous suture pattern should be applied first to bring skin edges into apposition [50]. In the present study subcutaneous suture was performed first to minimize tension at the wound margins, and special care was taken to place staples correctly. However, tissue strangulation and cross-scar formation were not prevented.

Cross scar formation was the main disadvantage of staples; otherwise, their application was very quick, not technically demanding and followed by a good cosmetic outcome. Furthermore, the fact that the marks fade with time and become less visible 70 days po. makes cross-scaring of minor importance. Anate [51], Orozco-Covarrubias and Ruiz-Maldonado [9] and Parell and Becker [52] also observed cross scar formation after a simple interrupted suture pattern, which faded after the 90th po.d.

#### 4.3.9. Total Clinical Evaluation

It is obvious that each technique influences wound healing differently. Some parameters of clinical evaluation are favorable for one technique while others are favorable for another. In the present study, in order to have an overall view of the total clinical evaluation of each technique, some of the scores of clinical parameters were summed up and a total clinical score for each one of the five periods was calculated.

As was expected, total clinical scores improved over time with all techniques. In period A, the improvement was larger with the intradermal suture pattern. On the contrary, with staples and glue, the clinical evaluation improved after period C.

In period A, the total clinical score of intradermal was better than those of staples and glue due to the milder skin thickening, the thinner scar and the smaller erythema at the wound area.

In period B, the total clinical score of intradermal continued to be better than those of glue and staples due to the narrow scar width and the mild skin thickening. Furthermore, during period B, the score of staples was statistically different from that of the other techniques, due to the cross-scaring formation and the intense skin thickening at the wound area.

In period C, the intradermal suture pattern was still the best. Moreover, staples had a statistically significant worst score compared to intradermal, as cross-scaring formations persisted.

In period D, the intradermal suture pattern had a better total clinical score compared to glue and staples, the difference being significant compared to staples. Although there were no cross-scaring formations, the larger scar width and the more pronounced scar pigmentation worsened the score of staples in comparison to intradermal, in which the scars were thinner with lower or absent pigmentation.

Finally, in period E, the total clinical score of intradermal continued to be better in comparison to those of staples and glue, the difference being significant between intradermal and glue. This change observed during the mature phase between staples and glue was due to the improvement of both width and pigmentation of the scar with staples in comparison to glue, with which the scar width continued to be large and with pigmentation.

Gouletsou et al. [4,5] also compared the total clinical evaluation between the intradermal suture technique pattern with burying the knots using absorbable monofilament sutures of different diameters and materials (4-0 poliglecaprone 25, 3-0 poliglecaprone 25 and 4-0 polyglytone 6211) and the simple interrupted suture pattern using 3-0 poliglecaprone 25, in surgical skin incisions in dogs. The intradermal techniques performed with 4-0 suture materials (poliglecaprone 25 and polyglytone 6211) were evaluated clinically as the best from the 3rd po. month until three years po.

### 4.4. Ultrasonographic Evaluation

In order to evaluate ultrasonographically the skin healing process over time, B-mode cutaneous two-dimensional sonograms of the wound area were performed and the wound area was estimated. As wound healing proceeded, the wound area size and the tissue oedema diminished, and the progressive collagen deposition altered echo intensity, making wound boundaries more complex but not indistinguishable.

In period A, the wound area was u/s estimated to be smaller with intradermal compared to the other techniques. Staples and glue had the largest values of the wound area, something that was in accordance with the larger skin thickening observed clinically. Obviously, during this period the dominant characteristic of the wound was oedema, the intensity of which depended on the technique used.

In period B, although the wound area was substantially decreased with all techniques, it was u/s estimated to be smaller with staples in comparison to glue and intradermal, the difference between intradermal and staples being significant. During that time period, the removal of staples reduced oedema, whilst intradermal continued to produce tissue irritation. From the middle of this period, the wound area with all techniques was reduced almost by half compared to the initial measurements.

In period C, wound size was even more decreased with all techniques and was u/s estimated to be smaller with staples. A statistically significant difference was revealed only between staples and intradermal.

In period D, a statistically significant difference between the techniques was not revealed. During this period, and specifically up to the 120th po.d., although the u/s wound area was small with intradermal and glue, it was yet distinctly imaged, due to the hypoechoic appearance of the newly formed dermis. On the contrary, it was imaged with difficulty with staples, because the echogenicity of the wound area was similar to the adjacent normal skin. After the 150th po.d., in almost all incisions with all techniques, the wound area was imaged as a small and low-sound-intensity area inside the healthy echogenic dermis and below the more echogenic junction of the epidermal edges.

In period E, although the u/s measured wound area was minimal with all techniques, it was estimated as smaller with staples in comparison to intradermal and glue; however, a statistically significant difference between the techniques was not revealed, while some difference was observed between staples and glue (*p* = 0.03).

Generally, from the 9th until the 365th po.d., a smaller wound area was constantly recorded with staples, probably because the absence of suture material inside the wound area in this technique led to less tissue reaction. On the contrary, with intradermal, the concurrent tissue reaction to suture material present in the wound area, up to the 119th po.d., contributed to the larger wound area. As regards tissue glue, the loose apposition of the wound edges might have contributed to the larger wound area. It should also be mentioned that on the 365th po.d., the wound area could not be detected in a few segments of some wounds with all techniques, suggesting complete skin repair.

In conclusion, as far as u/s estimated surgical wound size is concerned, the technique that had a small wound area over all time periods was intradermal, while staples had the largest wound area during the first week but changed to the have the smallest until the end of the experiment.

The correlation test between u/s estimated wound area and clinically evaluated scar width showed that there is a statistically significant linear positive correlation between the two parameters, i.e., the larger the u/s estimated wound area, the larger the scar width measured by electronic calipers on the surface of the skin. However, one can say that ultrasonographic measurement of a scar area is favorable, as it evaluates the extent of the scar even when a scab covers the wound. Also, ultrasonography depicts the extent of the scar under the surface of the epidermis, which is the only visible part during a clinical examination. Furthermore, the correlation test between u/s estimated wound area at the biopsy site and histologically estimated inflammatory reaction at the same area showed that there is a statistically significant positive correlation between the two parameters. This means that the larger edema and leucocyte infiltration due to inflammatory reaction at the wound area, the larger the area with reduced echogenicity calculated ultrasonographically. More interestingly, ultrasonography was able to reveal an epithelial gap in some cases, a finding that was not noticed by clinical examination but was confirmed by histological examination.

Based on all the above, it can be concluded that high-frequency ultrasonography can be used to evaluate the first intention healing of the canine skin. Ultrasonographic findings correlated very well with the clinical and histological findings and depicted the real ultrastructure of the skin during first intention healing.

### 4.5. Histological Evaluation

The histological examination gives detailed information on the healing process of the traumatized skin that may not be revealed by any other method. However, in clinical settings where animals or humans are used, performing multiple skin biopsies during healing is very difficult or even unfeasible. As a result, very few studies that employ histological examination of skin healing may be found in the bibliography [6], and even fewer were performed on canine skin [4,5].

#### 4.5.1. Edema

Edema was a constant finding on the 7th po.d. When edema was observed on the 14th or 28th po.d., it was accompanied by inflammation at the wound area. Gouletsou et al. [4,5] also found edema in almost all wounds examined on the 7th po.d. with all the techniques they employed, in 17/40 samples on the 14th po.d. and in 5/40 samples on the 28th po.d. In their study, all the cases of edema on the 28th po.d. were found in samples where inflammation was also present [4,5]. Since they gave no antibiotics, inflammation and edema were more common and lasted a longer period of time [4,5]. Papazoglou et al. [6] evaluated edema in six cats subjected to skin suturing with intradermal suture pattern with either the copolymer of glycolide, ε-caprolacton and trimethylene-carbonate or with polypropylene suture with clips. In their study edema was present in all incisions sutured with the first suture οn the 2nd po.d., but subsided οn the 7th po.d. In contrast, swelling of incisions was present in three cats sutured with polypropylene οn the 2nd po.d., but subsided in all cats οn the 7th po.d.

#### 4.5.2. Inflammation

No statistically significant difference in inflammation score was observed between the techniques. However, more cases of mild or severe inflammation were observed with intradermal (21 cases) and with glue (20 cases) than with staples (18 cases).

Skin stapling caused minimal or mild inflammation at the incision area. It seems that the use of inert metal staples that are removed 10 days po.d. minimizes foreign body inflammatory reaction at the wound area. Skin glue application caused mild to medium inflammatory reactions to the wound area. The most important finding was that in spite of care during the application, small quantities of glue were deposited below the epidermis, initiating inflammation in the dermis, a finding that persisted until the 365th po.d. According to Mobley et al. [53], glue implanted subcutaneously may induce chronic inflammation including edema, erythema, pain, or purulent drainage. Toriumi et al. [54] demonstrated significant inflammation and even tissue necrosis attributed to exposure of butyl-2-cyanoacrylate to well-vascularized subcutaneous tissues. In contrast, subcutaneous placement of the adhesive within the confines of two cartilage surfaces without exposure to the surrounding vascular tissue failed to elicit a significant inflammatory response. Toriumi et al. [54] also showed that inadvertent use of this agent below the skin and in unprotected subcutaneous tissue can result in moderate tissue damage and even necrosis. According to the above-mentioned, insertion of the glue into the subcutaneous layers may potentially increase the chance of delayed wound healing or inflammation and necrosis. However, in the present study, the inflammatory reaction around inclusions was of only 3–6 layers of cells, so it seems that their small dimensions could not substantially affect wound healing. Furthermore, no necrosis, intense inflammation or purulent drainage was noticed.

With the intradermal, the first postoperative month, the main finding was an infiltration of the wound area by a moderate or large number of neutrophils, macrophages and fibroblasts. Minimal to mild tissue reaction was usually observed around the suture material. It seems that the presence of suture material for a long time postoperatively enhances the presence of leucocytes in the wound area, as it promotes a foreign body reaction. However, no statistically significant difference was observed between this technique and the others that no material was implanted. Gouletsou et al. [5] also observed that moderate to large numbers of neutrophils and macrophages infiltrated the wound area during the first 14 days postoperatively when the wound was sutured by intradermal suture pattern with burying of the knot. However, in their study, more cases of severe inflammation (perivascular, nodular or diffuse) were detected, probably because no antibiotics were administered postoperatively.

Papazoglou et al. [6] evaluated inflammation in six cats following suturing of the skin with intradermal suture pattern either with a copolymer of glycolide, ε-caprolactone and trimethylene-carbonate sutures or with polypropylene suture with clips and noticed that cellular reaction was moderate to severe in most incisions, regardless of the closure technique. This might be due to the trauma produced by the placement of the needle and due to the reaction to the suture material.

#### 4.5.3. Epidermal Thickness

During the first po. days in the present study, the epidermal thickness at the wound area was increased in relation to the adjacent healthy skin with all techniques. Increased epidermal thickness was not restricted to the newly formed epithelium but was also extended to the intact wound edges. The intense proliferation of cells at wound borders is intended to replace losses due to the continued movement of epidermic cells to the epidermal deficit [12].

In period A, the median thickness of the epidermis at the area of wound healing was approximately 2.5 times that of the adjacent healthy epidermis with staples and intradermal whilst it was 3.5 with glue. The same, but to a lesser extent, was observed in periods B and C. No difference was observed from period D onwards, as the thickness of the epidermis at the wound area was almost similar to that of the normal adjacent skin. It seems that staples and intradermal have the same impact on the epithelial thickness at the wound area and that the initially increased epithelial thickness subsides one month postoperatively. On the contrary, with glue, the increase in epithelial thickness is significantly higher than that of the other techniques and lasts for a longer period of time. It is possible that the presence of glue substance at the wound area provoked increased proliferation of the epidermis at wound borders, in an effort of the epithelium for optimal epithelialization. It is also possible that chemical or thermal irritation of the epidermis due to polymerization of the glue resulted in an intense increase in epithelial thickness at the wound area, so a longer time period was needed in order to restore to normal.

Papazoglou et al. [6], observed that on the 9th po.d the thickness was larger in four out of six sections which included glycolide, ε-caprolactone and trimethylene-carbonate, whilst it was normal in all sections which included polypropylene. However, the method of measuring epithelial thickness differs between this study and that of Papazoglou et al. [6]. Furthermore, species differences may contribute to this discrepancy.

Gouletsou et al. [4,5] also observed two- to three-fold increases in epithelial thickness on the 7th po.d with all the techniques used, and in some occasions it reached a five-fold increase. The increase in their study subsided on the 28th po.d and was smaller in incisions closed by intradermal technique.

#### 4.5.4. Epithelial Gap

An epithelial gap was observed in only three samples with glue on the 7th po.d., while epithelial bridging had been completed on the 14th po.d. in all samples. It seems that the use of glue may delay epithelial bridging, probably due to the deposit of small quantities of glue below the epidermis, especially when the edges of the wound are not in close contact during glue application. This may initiate inflammation of the dermis or delay the migration of the cells of the epidermis. Gouletsou et al. [4,5] observed that epithelial bridging was delayed more and that 25 out of 40 samples from all the techniques they used presented epithelial gap on the 7th po.d. Perhaps in the present study, the use of antibiotics postoperatively contributed to the faster epithelialization observed in all techniques. Pope [55] also found that epithelialization was completed in the first postoperative days after surgical skin closure. Kirpensteijn et al. [2], who checked the epithelial bridging after suturing skin incisions with either intradermal suture with poliglecaprone 25 or polyglactin 910, also observed that all the wounds had been epithelialized at the 7th po.d.; however, they give no information about antibiotic administration.

Papazoglou et al. [6] noticed that epithelial bridging was completed on the 9th po.d. in all incisions closed with polypropylene but in only one out of six incisions closed with glycolide, ε-caprolactone and trimethylene-carbonate suture.

#### 4.5.5. Histological Scar Width

No significant differences were revealed between the techniques in histologically evaluated scar width in any time period. It seems that scar width becomes narrower over time with all techniques; however, improvement was poorer with glue than with the other techniques. Furthermore, the scar width with glue was larger than that of the other techniques throughout the whole experimental time, indicating that glue should not be used when a narrow scar is mandatory.

The only other study of histologically evaluated scar width is that of Gouletsou et al. [5], which observed that scar width was larger in incisions sutured with simple interrupted stitches in comparison to those sutured with intradermal suture patterns. Their findings were constant for the whole study period, which was three years postoperatively. In that study, the intradermal suture pattern with burying of the knots produced a scar of approximately 0.7 mm in width, which is slightly larger than the values found in the present study, i.e., 0.45–0.56 mm. This difference may be partly the result of antibiotics administration and the less intense inflammation observed in the present study.

#### 4.5.6. Fibroblasts Presence, Collagen Deposition, and Angiogenesis

In all periods, the presence of fibroblasts, collagen deposition and angiogenesis were not significantly different between the techniques.

Gouletsou et al. [4,5] observed that collagen fibers in the healing area were thinner than that of the adjacent skin even three years postoperatively. Van Winkle et al. [56], who measured biochemically the collagen quantity in skin wounds closed with different suture materials, observed that collagen production was not affected by the type of suture material. Gouletsou [4,5] also found no difference in the number of fibroblasts, collagen production and angiogenesis between the techniques used. Papazoglou et al. [6], when evaluating wound vascularity and collagen content in six cats after suturing their skin with intradermal suture pattern either with copolymer of glycolide, ε-caprolactone and trimethylene-carbonate or with polypropylene suture with clips, observed no significant difference between the two techniques. However, it is possible that the scoring system of collagen production, which was used in the present study and had been established in studies on skin healing by second intention, may not be suitable to estimate subtle differences in collagen production during wound healing by first intention. Furthermore, the use of more specific stains perhaps could give more information on collagen production.

#### 4.5.7. Total Histological Evaluation

The total histological evaluation for each time period was recorded after summing up the individual scores of edema, inflammation, thickness of the epidermis at the wound area, epithelial gap and scar width.

In period A, a better total histological score was recorded with staples in comparison to glue. The latter had the worse score due to larger scar width, larger epidermal thickness at the wound area and the presence of an epithelial gap in a few cases. In period B, a better total histological score was recorded with intradermal in comparison to staples and glue. In periods C and D, a better total histological score was recorded with staples and intradermal in comparison to glue. In period E, the total histological score was equal in all techniques.

Although no significant differences were observed between the techniques, in relation to collagen deposition, fibroblasts’ presence, angiogenesis and inflammation, the total histological score of the incisions closed with glue was worse until the 160th day. Delayed epithelialization in a few cases and wider scar affected negatively the total histological score with glue in comparison to staples and intradermal suture pattern. Intradermal suture pattern was better due to optimal epithelialization and a smaller scar. However, on the 365th po.d., when the only histological finding is scar width, since all the rest subside, no histological differences can be observed between the techniques.

### 4.6. Total Evaluation

After summing the scores of cosmetic, clinical, ultrasonographic and histological evaluation in each time period, the total score of each technique was calculated, in an attempt to estimate the overall depiction of each technique over time. The distinctive findings of each parameter with each technique are important; however, a clinician has to choose a closure technique that is overall best for the animal. Therefore, a total evaluation of each technique was attempted, even though the conclusions should be handled with some concern.

Thus, in period A, intradermal presented the best total score, followed by staples, and lastly, glue, which differed significantly from intradermal. It seems that during the first postoperative week, the intradermal technique accomplishes better contact of skin edges and allows faster epithelialization, and therefore a better overall result is achieved.

In period B, intradermal presented the best total score, followed by glue, and lastly, staples.

In period C, intradermal presented the best total score, followed by staples, and lastly, glue. One month postoperatively, the intradermal suture pattern causes mechanical irritation of the healing area and foreign body inflammatory reaction. Gouletsou et al. [4,5] also found that with intradermal suture patterns, the good cosmetic outcome observed during the first postoperative period deteriorates during the 4th-8th postoperative weeks and improves again afterwards, when the suture material is absorbed. However, the intradermal suture patterns in general have a better overall score than the other two techniques.

In period D, intradermal presented the best total score, followed by staples, and lastly, glue. Six months postoperatively, inflammation, being the result of either the healing process or foreign body reaction, had subsided, as the poliglecaprone suture material had been absorbed two months ago. The intradermal suture pattern seems to have a better overall score than the other techniques, probably because it promotes better contact of skin edges and holds them together for a longer period of time, preventing the expansion of the scar.

Finally, in period E, intradermal still presented the best total score, followed by staples, and lastly, glue. However, no significant difference was revealed between any of them.

In the present study, the data revealed that the cosmetic, ultrasonographic, clinical and histological results were better with intradermal suture pattern. Furthermore, the results with tissue glue were worse than the other techniques. A possible explanation for this is that tissue glue sloughs off with the shedding of epidermal cells and therefore cannot provide long-term approximation of wound edges so that adequate wound healing is achieved. Furthermore, when wound edges are not approximated well enough to prevent tissue glue from entering the wound, inflammation may be induced. Staples achieved good results with an easy and quick application and should be considered a sufficing method of wound closure in dogs.

## 5. Conclusions

With all techniques, the cosmetic outcome improved over time until the 63rd po.d., but afterwards the improvement was minimal. From the 9th po.d onwards, glue application had the worst cosmetic outcome compared to the other techniques. Staples induced the largest skin thickening in the wound area in the first postoperative days until staple removal, and then, from the 12th po.d. onwards this technique showed the least skin thickening. Tissue glue application induced the largest skin thickening from the 10th until the 365th po.d. Scar width decreased over time with all techniques. Scar width was larger with tissue glue than with the other techniques from the 9th po.d. onwards and was significantly larger than intradermal suture pattern on the 365th po.d. The total clinical evaluation was constantly better with intradermal suture pattern compared to the other two techniques. High-frequency ultrasonography was found to be effective for evaluating first intention healing of the canine skin. The total histological score was worse with glue in all time periods. Histological examination showed that, in spite of care in application, small quantities of glue penetrated the wound, causing inflammation in the dermis. Total evaluation of each technique revealed that the intradermal suture pattern had the best score throughout the experiment period, with a statistically significant difference from glue in the first postoperative week. Tissue glue application consistently had the worst total score, with no statistically significant difference after the first week.

If the best scar is the goal of first intention healing, an intradermal suture pattern is the best option. However, when the time required for skin closure is an important factor for patient management, or when there is a long incision, stapling is considered the first choice. Tissue adhesive is the least desirable option when considering overall wound healing.

## Figures and Tables

**Table 1:** Time (minutes/seconds) required for completion of each technique, all the differences being significant (*p* < 0.001).

Technique	Percentile 25	Median	Percentile 75	Mean	Standard Deviation
Staples	18 s	21 s	25 s	21 s	5 s
Glue	1 min 45 s	2 min 16 s	2 min 32 s	2 min 12 s	37 s
Intradermal	15 min 32 s	15 min 37 s	16 min 55 s	16 min 13 s	1 min 30 s

**Table 2:** Cases of inflammation for each technique.

	Score	Staples	Glue	Intradermal
Cases of inflammation	1	5	9	14
	2	0	0	1
Total		5 (3) *	9 (4)	15 (3)

* In brackets is the number of animals that showed inflammation.

**Table 3:** Incidence of each inflammation score for each technique.

Inflammation Score	Staples	Glue	Intradermal
0	19	18	20
1	13	12	9
2	12	12	14
3	6	8	7

**Table 4:** *p* values of comparisons between techniques for total evaluation.

Technique	Period	Glue	Intradermal
**Staples**	A	0.165	0.165
	B	0.853	0.043
	C	0.436	0.436
	D	0.353	0.143
	E	0.156	0.549
**Glue**	A		**0.007**
	B		0.075
	C		0.190
	D		0.143
	E		0.075

## Data Availability

The data presented in this study are available are available from the corresponding author on reasonable request.

## Supplementary Materials

The following supporting information can be downloaded at: https://www.mdpi.com/article/10.3390/ani13030426/s1, Figure S1: Photographs of the wounds; Figure S2: Ultrasound scans of the wound area; Figure S3: Photographs of the histological sections of the wound area; Table S1: Scoring system of clinical examination. Table S2: Scoring system of histological examination.
