# Supplementary material for: Evaluation of Incisional Wound Healing in Dogs after Closure with Staples or Tissue Glue and Comparison to Intradermal Suture Pattern
Source: Animals (Basel). 2023 Jan 27;13(3):426. doi: 10.3390/ani13030426 (PMC9913468; doi:10.3390/ani13030426)
Supplement: Supplementary file 1 [file animals-13-00426-s001.zip › animals-2168342-supplementary.pdf]

Supplementary Files

Figure S1: Photographs of the wounds

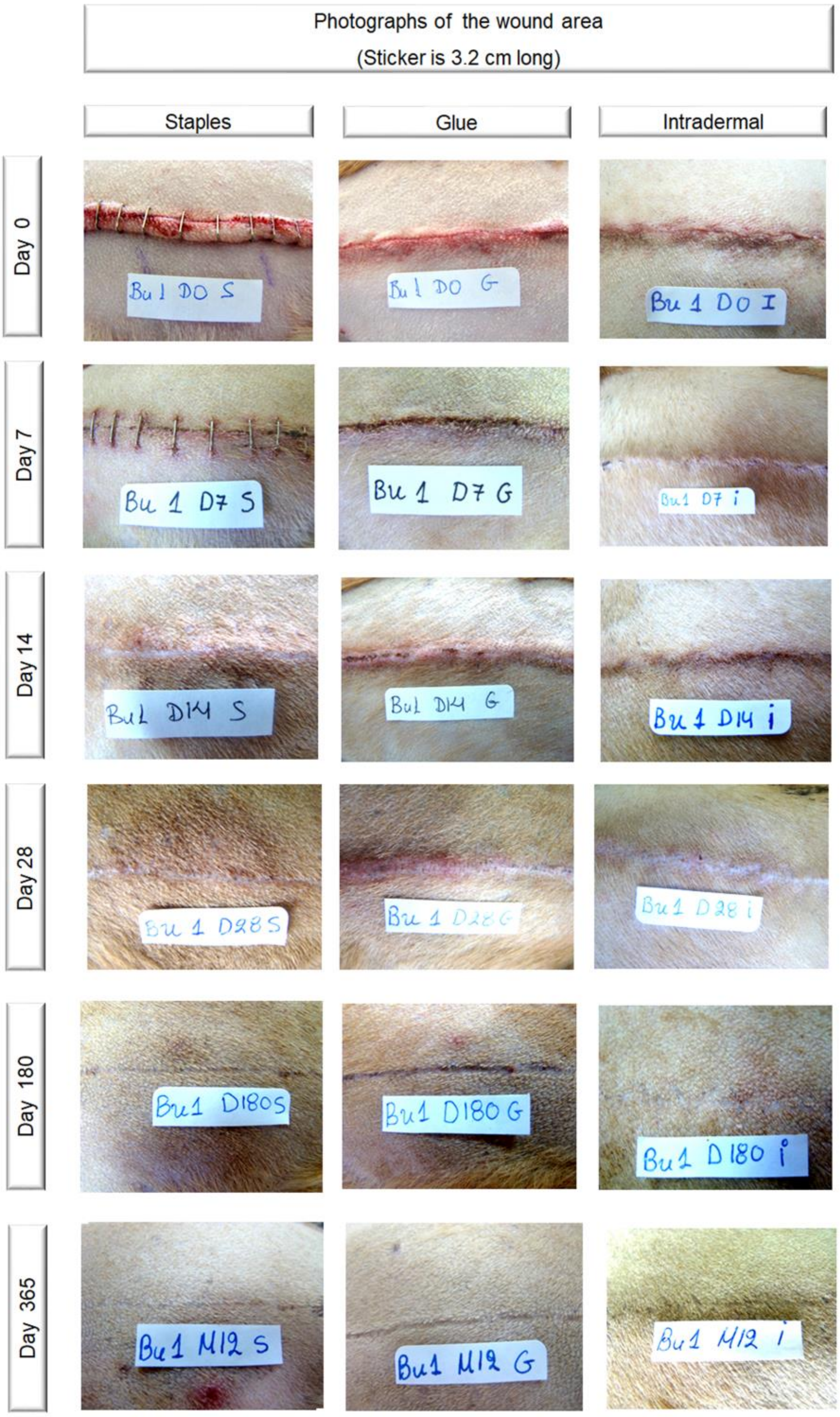

Figure S2: Ultrasound scans of the wound area

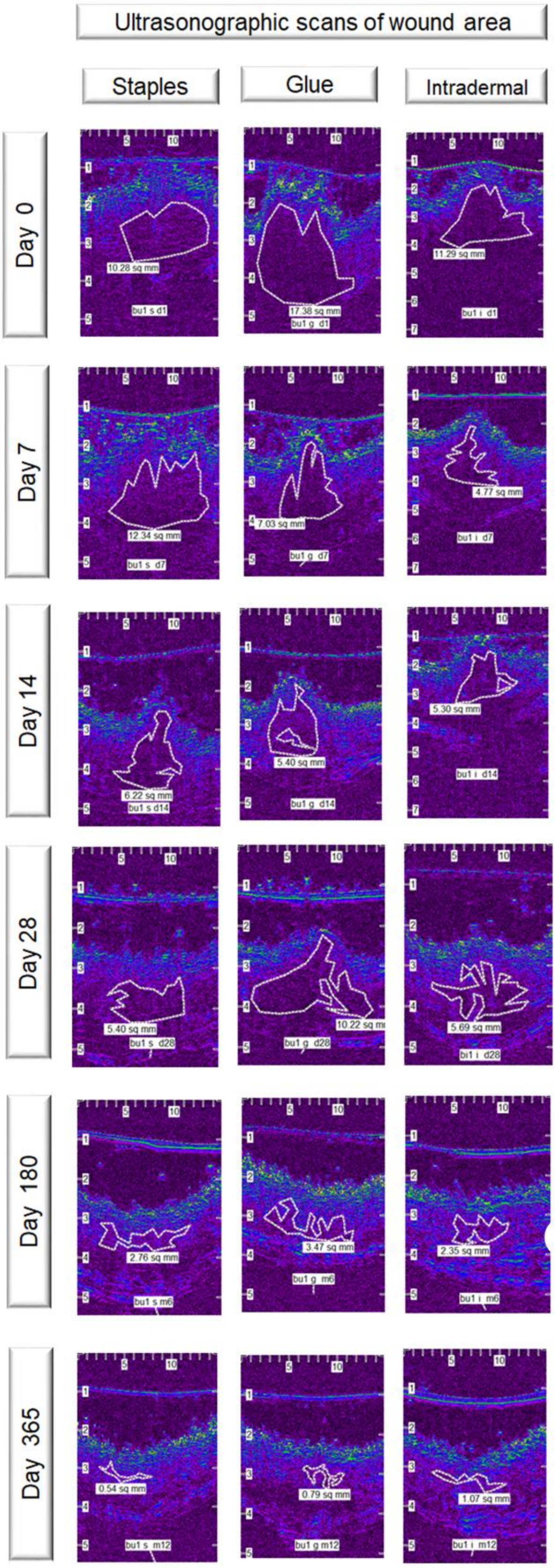

Figure S3: Photographs of the histological sections of the wound area 9

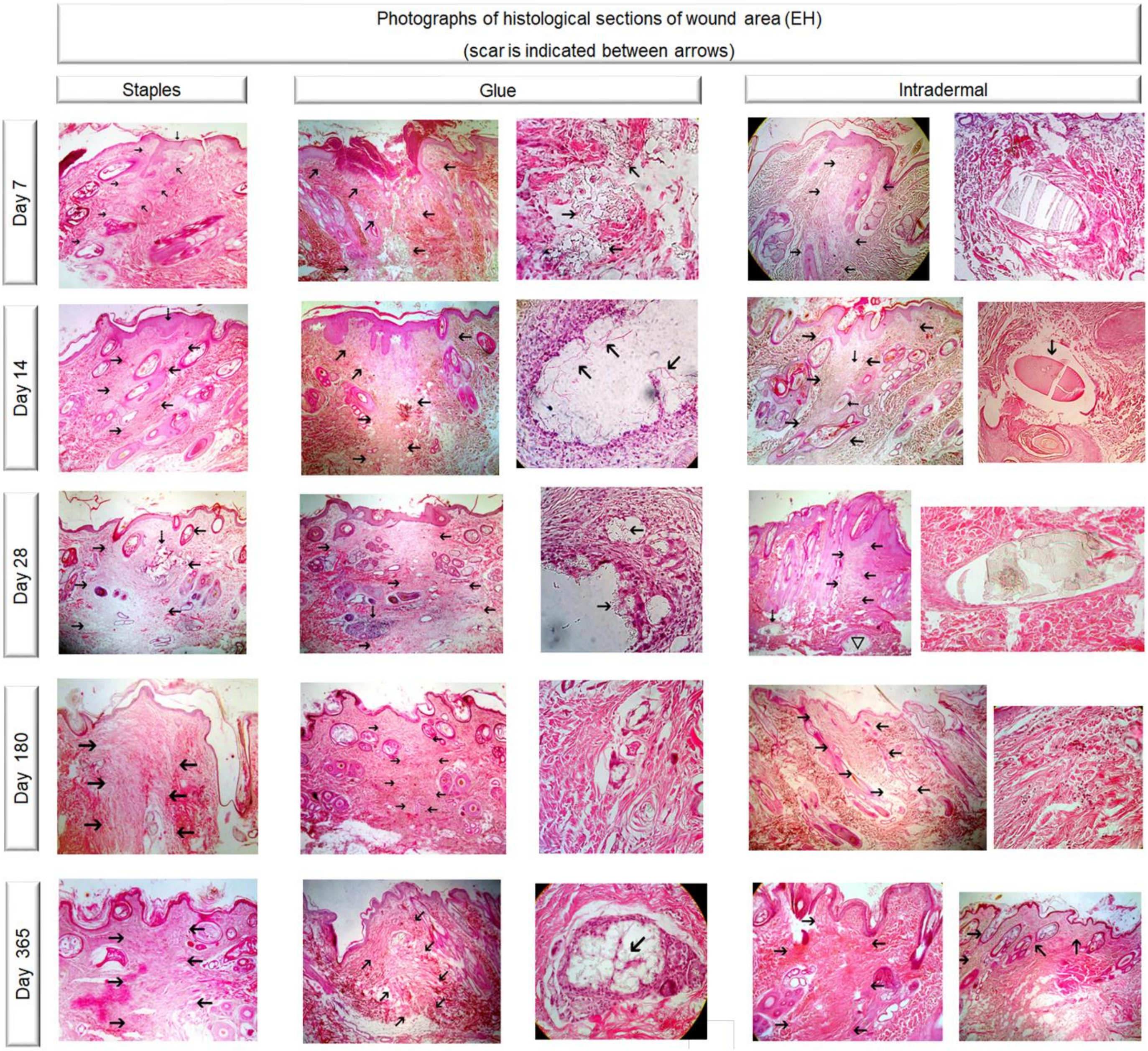

10

11

12

Table S1: Scoring system of clinical examination.

**CLINICAL EVALUATION**	**Score 0**	**Score 1**	**Score 2**	**Score 3**
Skin thickening	measured with a skin caliper at the central part of the incision, in mm			
Erythema	width of skin redness at the central part of the incision, measured with an electronic caliper, in mm			
Scar width	measured with an electronic caliper, in the central part of the incision, in mm			
Abscessation or inflammation	absence	mild inflammation	1-2 microabscesses	intense inflammation, or more than 3 microabscesses
Exudate	absence	serosanguineous	seropurulent	purulent
Comedones	absence	1-3	4-6	more than 7
Hyperpigmentation of the wound area, compared with the adjacent skin color	absence	mild	moderate	intense
Suture or staples loss	number of staples removed			
Wound dehiscence	Length in cm			
Cross-scaring marks	absence	1-2 marks	3-6 marks	more than 7

Table S2: Scoring system of histological examination.

HISTOLOGICAL EVALUATION	Score 0	Score 1	Score 2	Score 3
Necrosis [23]	none identified	few scattered areas	multiple focal dense areas	necrosis present throughout the slide
Epithelial gap [5]	mm			
Oedema [22]	absence of oedema, normal,	slight separation of cells and collagen from each other in the wound tissue by non-stained or poorly stained acellular material	separation of approximately 30 to 50 µm by this acellular material,	separation of > 50 µm)
Inflammation [2]	<3 cells/field	3-10 cells/field	11-30 cells/field	>31 cells/field
	evaluated by scoring neutrophils, eosinophils, macrophages, lymphocytes, plasma cells, and mast cells detected in 10 high power fields (HPF) (400x)			
Presence of suture [5]	absence of suture material	presence of a small part of the suture material,	presence of large part of the suture material,	presence of the entire suture material
Tissue reaction around the suture [2]	0-2 cell layers around suture tract	3-5 cell layers around suture tract	6-10 cell layers around suture tract	>10 cell layers around suture tract
Epithelial thickness [2]	x normal epithelial thickness			
Scar width [5[	mm			
Collagen synthesis [22, 23]	no collagen	scant collagen bundles slightly separating fibroblasts	dense accumulations of collagen between fibroblasts	extensive separation of fibroblasts by collagen
Presence of fibroblasts [22, 23]	<3 fibroblasts/field 400x	3-10 fibroblasts/field 400x	11-30 fibroblasts/field 400x	>31 fibroblasts/field 400x
Angiogenesis [22,23]	<3 capillary buds/field 400x	3-10 capillary buds/field 400x	11-30 capillary buds/field 400x	>31 capillary buds/field 400x

[2] Kirpensteijn, J.; Maarschalkerweerd, R.J.; Koeman, J.P.; Kooistra, H.S.; van Sluijs, F.J. Comparison of two suture materials for intradermal skin closure in dogs. *Vet Q* **1997**, *19*, 20-22, doi:10.1080/01652176.1997.9694732.

[5] Gouletsou, P.G.; Prassinos, N.N.; Papazoglou, L.G.; Kostoulas, P.; Galatos, A.D. Comparison of continuous intradermal with simple interrupted suture pattern: an experimental study in dogs. *Top Companion Anim Med* **2020**, *41*, 100454, doi:10.1016/j.tcam.2020.100454.

[22] Gillette, R.L.; Swaim, S.F.; Sartin, E.A.; Bradley, D.M.; Coolman, S.L. Effects of a bioactive glass on healing of closed skin wounds in dogs. *Am J Vet Res* **2001**, *62*, 1149-1153, doi:10.2460/ajvr.2001.62.1149.

[23] Winkler, J.T.; Swaim, S.F.; Sartin, E.A.; Henderson, R.A.; Welch, J.A. The effect of a porcine-derived small intestinal submucosa product on wounds with exposed bone in dogs. *Vet Surg* **2002**, *31*, 541-551, doi:10.1053/jvet.2002.34669.
